# Genomic and Proteolytic Profiling of *Lacticaseibacillus* sp. PRA205: Insights into PepX-Mediated Bioactive Peptide Metabolism

**DOI:** 10.1007/s12602-025-10879-7

**Published:** 2025-12-23

**Authors:** Marianna Cristofolini, Alice Cattivelli, Alessandra Barbieri, Giulia Zaccarini, Loris Bertoldi, Lisa Solieri, Davide Tagliazucchi

**Affiliations:** 1 Lactic acid bacteria and Yeast Biotechnology Lab (LYB), Department of Life Science, University of Modena and Reggio Emilia, Reggio Emilia, 42122 Italy; 2 Nutritional Biochemistry Lab, Department of Life Science, University of Modena and Reggio Emilia, Reggio Emilia, 42122 Italy; 3 BMR Genomics Srl, Padua, 35131 Italy

**Keywords:** Proteolytic system, X-prolyl dipeptidyl aminopeptidase, *PepX* gene, Lacticaseibacillus, Probiotics, Bioactive peptides

## Abstract

Species of the *Lacticaseibacillus casei* group are GRAS organisms extensively used in dairy fermentations to release bioactive peptides (BPs) with health-promoting properties. However, the genetic basis of this proteolytic phenotype remains poorly investigated. In this study, we characterised the probiotic features, biosafety, and proteolytic system of the probiotic candidate *Lacticaseibacillus* sp. PRA205, a high producer of the anti-hypertensive peptides Val-Pro-Pro (VPP) and Ile-Pro-Pro (IPP), originally isolated from Parmigiano Reggiano cheese, with a focus on the PepX enzyme. Whole-genome sequencing revealed a 3.2 Mb genome encoding 2977 predicted genes. Phylogenomics assigned PRA205, previously identified as *Lcb. casei*, to a lineage closely related yet distinct from *Lacticaseibacillus parahuelsenbergensis*. Genome annotation identified adhesion and stress-tolerance genes, along with three bacteriocin clusters, but no antibiotic resistance or virulence determinants. PRA205 exhibited a complete proteolytic system consistent with its strong proteolytic phenotype, including two pathways for the degradation of proline-rich caseins (PepX/PepQ and PepI/PepP). Notably, unlike other proteolytic genes such as *prt*, the single *pepX* gene found in PRA205 was only weakly repressed under peptide–rich nitrogen conditions, suggesting a regulatory mechanism partially independent of amino acid availability. The enzyme, a monomeric serine protease of ~ 80 kDa, was partially purified and biochemically characterised. PepX degraded BPs, including the tripeptides VPP and IPP, and retained activity at 4 °C and pH of 4.0, supporting a dual role in BPs production and breakdown during dairy fermentation and storage. Overall, these findings elucidate the genetic basis of the proteolytic activity of PRA205 and support its safety and potential use in the development of functional dairy foods enriched in BPs.

**Supplementary Information:**

The online version contains supplementary material available at 10.1007/s12602-025-10879-7.

## Introduction

In the dairy industry, microbial fermentation is one of the oldest and most effective methods for developing functional foods [1–4]. In this context, lactic acid bacteria (LAB) serve as cell factories to produce biofunctional compounds, including milk protein-derived bioactive peptides (BPs) [5–7].

BPs are encrypted sequences within milk proteins that, once released as short peptides (2–40 amino acids), exhibit various biological activities, including antimicrobial, hypocholesterolemic, anti-hypertensive, antioxidant, anticancer, antithrombotic, osteogenic, immunomodulatory, and mineral-binding activities [6, 8]. Their activity depends on sequence and composition, with many peptides showing multifunctionality through multiple mechanisms [6, 8, 9]. Beyond biological effects, short peptides released by LAB also affect cheese maturation, influencing the texture and flavour of the final products [10]. The link between cheese bitterness and peptide fractions enriched in hydrophobic amino acids was first noted in 1932 [11]. Conversely, other peptides enhance the salty and umami notes of cheese by interacting with compounds like reducing sugars and unsaturated fatty acids, forming flavour molecules such as sulfur compounds, methyl ketones, and aldehydes [12]. In LAB, a coordinated proteolytic system hydrolyses milk proteins into peptides and amino acids, compensating for the autotrophies of these bacteria for several amino acids and enabling growth by utilising external proteins as a nitrogen source [13, 14]. This system includes cell surface proteinases (CEPs), that cleave caseins into oligopeptides (up to 17 amino acid residues); specific transporers for the peptide internalisation, such as Opp, DtpT, Dpp, and di/tri-peptide transporters; and a broad range of intracellular peptidases, including specific endopeptidases, aminopeptidases, tri- and dipeptidases, and proline-specific enzymes, which further degrade peptides into smaller units [15–17]. Recent probiogenomic studies have revealed considerable variability in the repertoire of proteolytic genes and associated regulatory elements, accounting for the high phenotypic variability observed at the species and strain level in the ability to hydrolyse milk proteins and release BPs [17–19].

*Lacticaseibacillus* sp. strain PRA205, isolated from Parmigiano Reggiano PDO cheese and previously identified as *Lacticaseibacillus casei* PRA205 [20], is a mesophilic, non-starter LAB showing tolerance to environmental cues such as acidity, salinity, bile salts, and digestive enzymes [21]. During milk fermentation, it produced notable levels of the anti-hypertensive tripeptides Valine-Proline-Proline (VPP) (32.88 mg/L) and Isoleucine-Proline-Proline (IPP) (7.52 mg/L), conferring strong angiotensin-converting enzyme (ACE) inhibitory activity [22, 23]. Moreover, when used as an adjunct culture during yoghurt fermentation with *Lactobacillus delbrueckii* subsp. *bulgaricus* and *Streptococcus thermophilus*, strain PRA205 maintained viability above 10⁸ CFU/g for 28 days at 4 °C, exceeding the minimum threshold required for probiotic efficacy [24].

In a previous work, the cell envelope proteinase (CEP)-encoding *prt* gene of strain PRA205 was characterised and the enzyme PrtR1 was identified as responsible for the release of several BPs through the hydrolysis of αs1- and β-caseins [25]. However, despite the probiotic potential of the strain, the entire proteolytic system of PRA205 remains uncharacterised, particularly the intracellular peptidases involved in peptide processing. Among these, X-prolyl dipeptidyl aminopeptidase (PepX, EC 3.4.14.11) is a serine protease belonging to the MEROPS peptidase family S15 (clan SC; http://www.ebi.ac.uk/interpro/entry/IPR008252), which cleaves Xaa-Pro bonds, releasing N-terminal dipeptides from polypeptides [26–28]. This aminopeptidase has been characterised in several LAB species but not in *Lacticaseibacillus* spp. In *Lactococcus lactis*, PepX shows a narrow specificity for proline (or for alanine or glycine with 10- and 100-fold lower efficiency, respectively) in the P1 position, while accepting any residue except proline at P2 and P′1 [27, 28]. Although the positive role of PepX in cheese peptide debittering is well-known, its contribution to the release of BPs remains debated. Several studies have linked PepX activity to notable biofunctions. PepX from *Lactobacillus helveticus* hydrolyses caseins [29, 30] and, together with PepC and PepO, contributes to the release of VPP and IPP from a β-casein precursor [31]. PepX from *Lactobacillus acidophilus* hydrolyses highly proline-rich gliadins and coeliac-toxic peptides [32–34], while PepX enzymes from five LAB species generate ACE-inhibitory peptides from goat milk [35]. Deletion of the *pepX* gene abolished hydrolysis of β-casomorphin-7 [36, 37]. Conversely, other studies found high PepX activity negatively correlated with BPs release. In milk fermented by *L. lactis* and *L. helveticus*, high PepX activity did not enhance ACE-inhibitory capacity, suggesting that ACE-inhibitory peptides were primarily generated by early-stage proteases [38]. Similarly, in *L. helveticus* CNRZ32, deletion of *pepX* and *pepN* increased ACE-inhibitory activity, likely due to reduced degradation of proline-rich BPs [15, 39].

This study aimed to explore the genome of the probiotic candidate *Lacticaseibacillus* sp. PRA205 and to investigate its safety and probiotic traits. We characterised its proteolytic system, focusing on PepX, to assess its biochemical properties in *Lacticaseibacillus* spp. Given the recent taxonomic revisions within this genus, including the introduction of four novel species [40, 41], a comparative genomic approach was employed to accurately determine the phylogenetic position of strain PRA205.

## Materials and Methods

### Reagents and Cultivation Media

Unless otherwise stated, media and anaerobic systems were purchased from Oxoid (Basingstoke, Hampshire, UK), while the chemicals were purchased from Sigma-Aldrich (St. Louis, MO, USA). Chemicals and solvents for mass spectrometry analysis were supplied by Carlo Erba (Milan, Italy). Primers were provided by BMR Genomics (Padova, Italy), the molecular biology reagents by Thermo Fisher Scientific (Waltham, MA, USA), and the Amicon Ultra-4 centrifugal filter units (regenerated cellulose, nominal cut 30 kDa) by Millipore (Milan, Italy).

### Bacteria Strain and Culture Conditions

The *Lacticaseibacillus* sp. strain PRA205, isolated from Parmigiano Reggiano PDO cheese [20], was deposited in the Culture Collection of the Department of Life Sciences (University of Modena and Reggio Emilia, Italy). The strain was cryo-preserved at −80 °C in the Man, Rogosa and Sharpe (MRS) medium (pH 6.5) containing 25% (v/v) of glycerol. Reactivation was performed in 5 mL of MRS medium, supplemented with 1.5% (w/v) agar when required, and incubated at 37 °C for 24 h, under anaerobic conditions.

### Reference Genomes

Genomes from *Lacticaseibacillus casei* group (LCG) species, including *Lacticaseibacillus casei*, *Lacticaseibacillus paracasei*, *Lacticaseibacillus rhamnosus*, *Lacticaseibacillus zeae*, and the four recently proposed species, such as *Lacticaseibacillus huelsenbergensis*, *Lacticaseibacillus zeae* subsp. *silagei*, *Lacticaseibacillus parahuelsenbergensis*, and *Lacticaseibacillus styriensis*, were used for comparative purposes and listed in Supplementary Table S1. In addition, two *Lactobacillus* species with a well-characterised proteolytic system, namely *Lactobacillus helveticus* and *Lactobacillus delbrueckii* subsp. *lactis* were used as reference genomes for reconstructing the proteolytic system. All the genomes were obtained from the National Center for Biotechnology Information (NCBI, Bethesda, Rockville, ML, USA) genome database (http://www.ncbi.nlm.nih.gov/genomes/lproks.cgi, last accessed 12 March 2025).

### Genomic Sequencing and Annotation

Genomic DNA was extracted as previously reported [42] and sequenced by BMR Genomics (Padua, Italy) using the Illumina MiSeq platform (2 × 300 bp). Reads preprocessing was carried out with Fastp v0.23.2 [43] to remove residual adapter sequences, short reads (length < 150), and low-quality data (base quality < 20, average read quality < 25, read complexity threshold < 30). Possible contaminants were assessed by MetaPhlan v4.0.1 [44].

SPAdes v3.15.5 [45] with the *careful* option was applied to perform *de novo* genome assembly. QUAST v5.0.2 [46] and BUSCO v5.4.3 [47], run on *lactobacillales_odb10* (v2020-03–06) lineage dataset, were used for assessing assembly metrics and genomic completeness, respectively. Gene prediction and annotation were performed with Prokka v.1.14.6 [48] and eggNOG-mapper v2.1.7 [49].

A custom graphic map of the genome was generated via Proksee v1.1.2 (https://proksee.ca; accessed 13 February 2025) [50] using the GenBank annotation file (gbk). BlastKOALA was used to calculate the relative abundances of genes in the KEGG categories as percentages of the genes assigned to each respective KEGG category versus the total genes number [51].

### Species Identification and Phylogenomics

Draft genome sequence of PRA205 and seven *Lacticaseibacillus* complete genomes were uploaded to the JspeciesWS server (https://www.ribocon.com/jspeciesws.html, accessed on 14 Mar 2025) to calculate ANI values with BLAST algorithm (ANIb) and MUMmer (Maximal Unique Match) alignment tool (ANIm), respectively [52]. FastANI, a k-mer and alignment-free method of ANI calculation [53], was implemented in the EDGAR3.0 webtool [54]. The calculations of average amino acid identity (AAI) and of the percentage of conserved proteins (POCP) between two genomes, as proposed by [55], were also implemented in the EDGAR3.0 pipeline [54]. For POCP analysis, the following cutoff values were used: an e-value threshold of 1*e*^− 5^, a minimum sequence identity > 50%, and an alignment coverage of the query protein > 50%.

GGDC 3.0 available in Type (Strain) Genome Server (TYGS) (https://tygs.dsmz.de/) was used to calculate digital DNA-DNA hybridisation (dDDH) values [56]. Phylogenomic analysis was carried out with two approaches. Firstly, the core genes of 8 genomes were computed in EDGAR3.0 pipeline [54]. The alignments of each core gene set are generated using MUSCLE [57], and the alignments are concatenated into one huge alignment. This alignment was the input for the FastTree software (http://www.microbesonline.org/fasttree/) to generate an approximately-maximum-likelihood (ML) phylogenetic tree. The values at the branches of the FastTree tree were local support values computed by FastTree using the Shimodaira-Hasegawa (SH) test.

Secondly, the PRA205 assembled genome was compared with 25 *Lacticaseibacillus* genomes (Supplementary Table S1). To perform such a procedure, PhyloPhlAn v3.1.68 [58] was applied in fast mode against a customised PhyloPhlAn database containing only *Lactobacillus*-associated markers (*n* = 339453). In addition, the following parameters were used to generate high-resolution strain-level phylogeny: --diversity low --trim greedy --min_num_entries 9 (~ 34.6% of the total number of genomes in order to keep more informative signals for distinguishing between very closely related organisms; 75481 markers were selected) --remove_fragmentary_entries --fast --force_nucleotides. The Maximum Likelihood (ML) tree was reconstructed from the PhyloPhlAn output using RAxML version 8.2.12 [59]. The GTRCAT model was applied to account for nucleotide substitution and rate heterogeneity. A rapid bootstrap analysis (-f a) with 1000 replicates was performed to assess branch support. To guarantee the reproducibility of the results, the parsimony random seed (-p) and the rapid bootstrap random seed (-x) were set to 1989 and 42, respectively.

All the resulting phylogenetic trees were further refined and visually enhanced using the iTOL tool [60].

### Genome Analyses

Comparative genomics, including core and accessory gene identification and functional categorisation (Orthologous Groups of proteins – COGs; KEGG orthologs - KO), were performed in EDGAR3.0 [54]. The carbohydrate-active enzyme (CAZy) database implemented in the ProbioMinServer web-platform was used for functional assignment of CAZy enzymes [61].

The biosynthetic gene clusters (BGCs) and bacteriocin-encoding genes were predicted with antiSMASH 4.0 [62] (https://antismash.secondarymetabolites.org/#!/about, accessed on 20 June 2025) and BAGEL5 (http://bagel5.molgenrug.nl, accessed on 20 May 2025).

Putative plasmids were identified using the PlasmidFinder v2.1 database (https://cge.food.dtu.dk/services/PlasmidFinder/) according to the following screening criteria: 95% identity threshold and 60% minimum coverage [63]. CRISPR (Clustered Regularly Interspaced Short Palindromic Repeats) sequences and Cas protein-encoding genes were identified using CRISPRCasFinder v2.2 [64]. The presence of genes of mobile elements was examined using BLASTX searches compared to the full mobileOG-db v1.1.3 database [65], with an identity > 90% and coverage > 90%.

The in silico analyses for microbial safety assessments were implemented in ProbioMinServer [61], according to the guidelines of the European Food Safety Authority (EFSA) [66]. Specifically, antibiotic resistance genes (ARGs) were identified using the Comprehensive Antibiotic Resistance Database (CARD) Variants v4.0.0 [67], ARMFinder [68], and ResFinder v4.3.2 [69]. BLASTN v2.8.1 + was used to detect virulence factors (VFs) by searching against the set B database from the Virulence Factor Database (VFDB) [70] and VirulenceFinder v2.0.3 [71]. BLASTP v2.8.1 + search was performed against the Pathogen Host Interaction v4.14 database to identify the probable pathogenic genes (PGs) [70] (Liu et al. 2022). The probiotic potential risk score (PPRS) was computed as defined by [72]. The score was classified as low-risk (≤ 4), medium-risk (4–6), and high-risk (≥ 6).

### Identification of Orthologs of the Proteolytic System

Protein sequences of experimentally verified proteolytic enzymes were obtained from Uniprot (http://www.uniprot.org/; accessed on 17 May 2025; [73]) (Supplementary Table S2) and used as queries for TBLASTN searches against selected genomes (Supplementary Table S1). Two sequences were considered homologous if their alignment had a minimum sequence identity of 30% and a query coverage of at least 70% [74]. Unannotated candidates were validated with HMMER v.3.2.1 using Pfam Hidden Markov Models (HMMs) profiles [75, 76].

### Milk Fermentation

Strain PRA205 was pre-cultured in 50 mL of MRS broth for 72 h at 37 °C under anaerobic conditions. After centrifugation at 10,000 rpm for 20 min at 4 °C, cells were washed with physiological solution (0.9% NaCl) and then inoculated in triplicate in scraw-cap flasks containing 45 mL UHT skimmed milk at the final concentration of 10^10^ CFU/mL. After 72 h of incubation at 37 °C under shaking conditions (10 rpm), fermented milk samples were left to settle for 10 min. The liquid phase was separated from the coagulated proteins through Whatman paper (grade 4). After centrifugation at 6,000 rpm for 20 min at 4 °C, cells were resuspended in 6 mL of 50 mmol/L phosphate buffer (pH 7.0) a final concentration of 10^10^ CFU/mL and divided into two aliquots for RNA extraction and biochemical characterisation of pepX enzyme, respectively.

### Gene Expression Analysis

RNA extraction and dsDNAase treatment were carried out as reported in the Supplementary Information. cDNA synthesis was performed using RevertAid Reverse Transcriptase (Cat. No. EP0441; Thermo Fisher Scientific) with both random hexamers (Cat. No. SO142; Thermo Fisher Scientific) and oligo (dT)18 primers (Cat. No. SO131; Thermo Fisher Scientific).

The end-point RT-PCR amplifications of the *pepX* gene and of the 16S rRNA gene (positive control) were carried out with a Dream Taq DNA polymerase (Cat. No. EP0712; Thermo Fisher Scientific). RT-qPCR reactions were done using the PowerUp SYBR Green Master Mix (Cat. No. A25742; Thermo Fisher Scientific) on a QuantStudio 3 real-time PCR system (Thermo Fisher Scientific, Waltham, MA, USA), with the 16S rRNA as the reference gene. Threshold cycle (Ct) values were analysed using QuantStudio Design and Analysis Software (v1.5.2). The measurement of gene expression was tested in biological triplicate, and the mean of values for the target gene were analysed and normalised to that of the 16 S rRNA gene using the 2^−ΔΔCT^ method [78]. Details on the reaction mixture were reported in the Supplementary Information.

All the primers used in this study are listed in Supplementary Table S3.

### Preparation of *Lacticaseibacillus* sp. PRA205 Cytoplasmic Extract

Induced cells obtained from milk fermentation were harvested (8,000 rpm, 10 min, 4 °C), washed 3 times with 100 mmol/L sodium phosphate buffer (pH 7.0), and resuspended in 20 mmol/L Tris-Cl buffer (pH 7.5; 1 mL of buffer per 10^10^ total cells). Cell disruption was performed by adding acid-washed glass beads (< 106 μm) (Cat. No. G4649; Sigma Aldrich) to the cell suspension in the proportion of 1:1 w/v and samples were vortexed at the maximum power with a Vortex-Genie 2 (Scientific Industries, Inc., Bohemia, NY, USA) for 4 min at 4 °C. The extraction step was repeated four times with 2 min of resting in ice after each vortexing cycle. At the end of the last cycle, cell debris and glass beads were removed by centrifugation for 40 min at 10,000 rpm at 4 °C. Finally, the supernatant, representing the cytoplasmic extract, was withdrawn, aliquoted and stored at −80 °C until further analyses [78].

Proteins in the cytoplasmic extract were quantified by the Bradford method using bovine serum albumin (BSA) as a standard [79]. The results were expressed in mg/L of BSA equivalents.

### Determination of PepX Activity in the Cytoplasmic Extract of *Lacticaseibacillus* sp. PRA205

PepX activity in the *Lcb. casei* PRA205 cytoplasmic extract was assessed in a 96-well plate using glycyl-prolyl-*p*-nitroanilide (Gly-Pro-*p*NA) as a specific substrate in the absence and presence of inhibitors [80]. The reaction mixture containing 235 µL of 50 mmol/L Tris-Cl buffer (pH 7) and 5 µL of Gly-Pro-*p*NA substrate (previously dissolved in the same buffer in a concentration of 6.4 mmol/L) was pre-incubated at 37 °C for 5 min, before the addition of 10 µL of cytoplasmic extract. The same reactions were also carried out in the presence of EDTA and PMSF at increasing concentrations of 0.1, 0.5, 1, 5 and 10 mmol/L. Controls contained a buffer instead of an extract. After incubation for 2 h at 37 °C, reactions were stopped with 50 µL of acetic acid (30%) and the release of *p*NA was measured spectrophotometrically at 405 nm.

The enzyme activity, defined as the amount of enzyme needed to release 1 µmol *p*NA per minute (U = µmol/min x mL), was calculated as in Eq. 1.

Eq. 1:

$$\:U=\left[\:\frac{\left(\left(\frac{\Delta\:Abs}t\right)\ast Vf\right)}{\left(\epsilon\:\:\ast Vc\right)}\:\right]$$

Where: ΔAbs is the absorbance change at 405 nm; t is the incubation time in minutes; Vf is the final reaction volume in mL; ε is the molar extinction coefficient of *p*NA, 0.00945; and Vc is the sample volume in mL. Finally, the specific enzyme activity was calculated by relating the units of enzyme activity to the protein content expressed in mg/mL, using the formula U/mg = µmol/min x mg.

### Partial Purification of PepX from the Cytoplasmic Extract of *Lacticaseibacillus* sp. PRA205

PepX was partially purified by using a size exclusion chromatographic column (Supplementary information). During all stages of the partial purification process, both the protein content and the specific activity of PepX were monitored using the methods described above. The cytoplasmic extract was first concentrated approximately 4-fold by ultrafiltration using 30 kDa filters and then subjected to size-exclusion chromatography using Sephadex G-100 resin. Active fractions were collected and further subjected to ultrafiltration with 30 kDa filters to concentrate the sample and remove the salts. The full protocol is reported in Supplementary Information.

### Effects of temperature, pH, and Inhibitors on PepX Activity

The effect of temperature on the PepX activity was determined as described above, modifying the incubation temperature while maintaining a constant pH of 7. The assay was carried out at four temperatures: 5 °C, 35 °C, 40 °C, and 45 °C. The effect of pH on enzyme activity was evaluated at a constant temperature of 37 °C by modifying the pH of the reaction buffer. Tris-HCL buffer (50 mmol/L) was utilized for reactions at pH 4–9, whereas sodium Tris-HCL buffer (50 mmol/L) was used for the assay at pH 7.0. The effect of protease inhibitors was tested by supplementing the reaction mix with EDTA or PMSF at final concentrations of 0, 1, 2, 5, or 10 mmol/L under standard conditions (pH 7.0, 37 °C).

### Peptide Degradation by Partially Purified PepX

The proteolytic activity of partially purified PepX was evaluated using the BPs IPP, VPP, LPPT, APFPE, IPPL, and PPF as substrates. Each reaction mixture contained 10 µL of enzyme and 90 µL of peptide solution, prepared by dissolving the peptide in 50 mmol/L Tris-HCl buffer (pH 7.0) to the final concentration of 0.5 mmol/L. Control reactions were also prepared by replacing the enzyme with 10 µL of potassium phosphate buffer (pH 7.5) containing 0.2 M NaCl, to assess potential spontaneous degradation of the peptides. Reactions were incubated at 37 °C for 24 h, and degradation products were analysed by a Q Exactive Hybrid Quadrupole-Orbitrap Mass Spectrometer (Thermo Scientific, San Jose, CA, USA) coupled to UHPLC system (UHPLC Ultimate 3000 separation module, Thermo Scientific, San Jose, CA, USA) equipped with a C18 column (Acquity UPLC HSS C18 reversed phase, 2.1 × 100 mm, 1.8 μm particle size, Waters, Milan, Italy), as described in [81].

The relative quantities of the peptides IPP, VPP, LPPT, APFPE, IPPL, and PPF were estimated by integrating the area under the corresponding peaks in the extracted ion chromatograms.

### Statistical Analyses

The data were displayed as the mean ± standard deviation (SD), with statistical significance denoted by *p* < 0.05. Comparative analysis between groups was conducted using a two-tailed unpaired Student’s t test and one-way ANOVA. All statistical assessments were carried out using Prism Graphpad 8.0 (GraphPad Software Inc.).

## Results and Discussion

### Genome Sequencing and Annotation

**Fig. 1:**
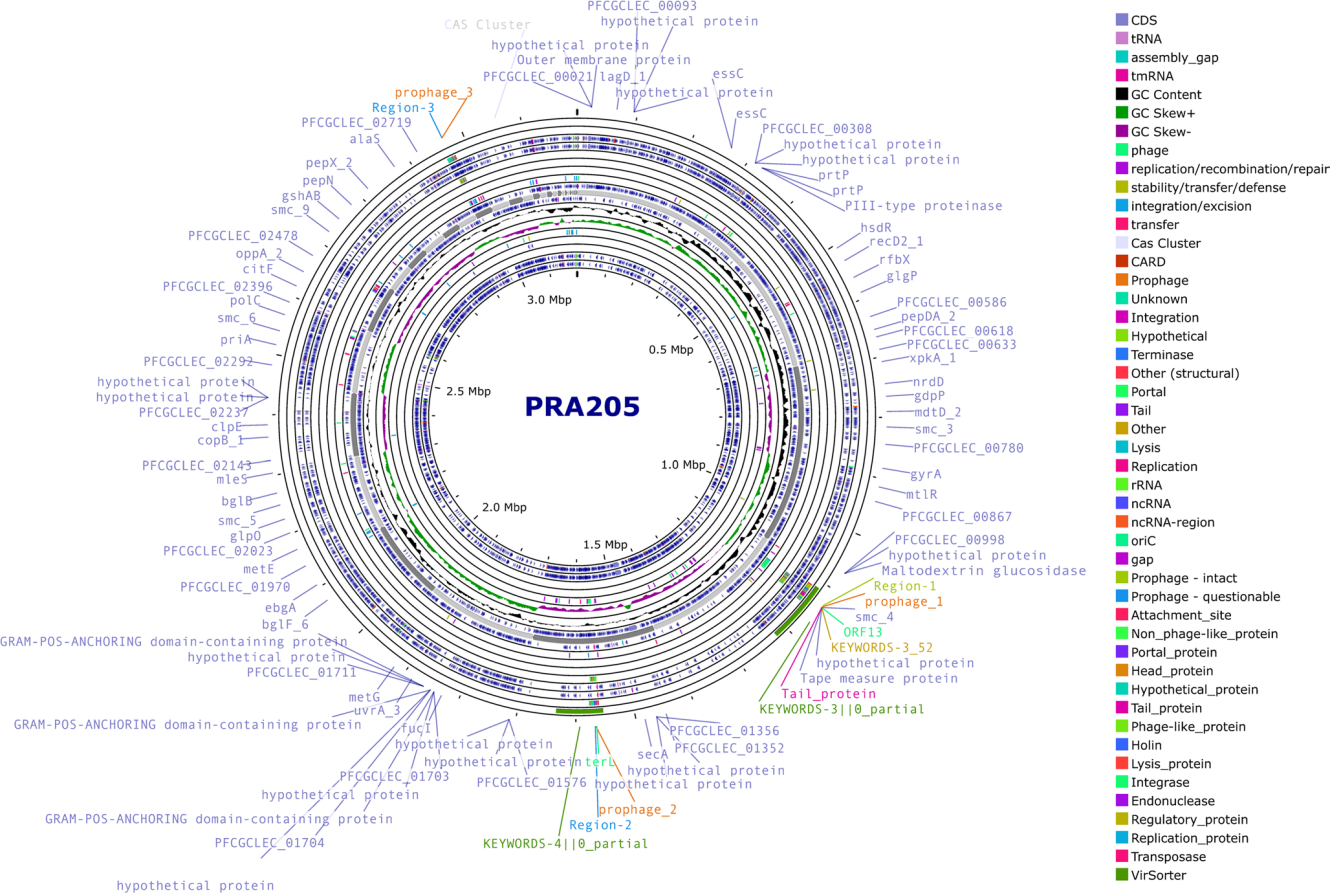
Circular graphical representation of *Lacticaseibacillus* sp. strain PRA205 contigs. The map of contigs was generated using Proksee (https://proksee.ca; accessed on 13 February 2025). The black central circle shows the scale expressed in megabases. Starting from the outermost ring: Ring 1: VirSorter, Ring 2: PHASTEST, Ring 3: Bakta Annotation (+), Ring 4: Prokka Annotation (+), Ring 5: Phigaro, Ring 6: CARD RGI Results (+), Ring 7: CRISPRCasFinder Annotation (+), Ring 8: mobileOG-db Annotation (+), Ring 9: Features (+), Backbone (Contigs), Ring 11: Features (-), Ring 12: GC Content, Ring 13: GC Skew, Ring 14: mobileOG-db Annotation (-), Ring 15: CRISPRCasFinder Annotation (-), Ring 16: CARD RGI Results (-), Ring 17: Prokka Annotation (-), Ring 18: Bakta Annotation (-). Selected genes are labelled on the outer purple ring using Proksee’s default settings. The GC content is in dark green, and the GC skew + and - in dark pink and dark green, respectively. The genomic order of the contigs is arbitrary

Strain PRA205 was characterised at the genome level. The assembly of 1,124,896 reads resulted in 38 contigs, corresponding to a total of 3,195,478 bp and 3038 features (2977 CDS, 54 tRNA, 6 rRNA, and 1 tmRNA; Table 1). The tRNAs, covering all 20 amino acids, were scattered across the contigs, with two clusters on contig 1 (772 bp, 8 tRNA) and contig 21 (∼2,293 bp,14 tRNA) (Fig. 1). The GC content was 47.82% and a clear definition of the positive and negative strands was obtained (Fig. 1).

**Table 1:** Assembly and annotation of *Lacticaseibacillus* sp. PRA205 genome

Assembly statistics	Features	Annotation statistics	Features
Contigs	38	Strand +	1323
Contigs (> 1000 bp)	33	Strand -	1709
Contigs (> 10.000 bp)	23	CDS	2977
Largest contig	684.487	tRNA	54
Total length	3.195.478	rRNA	6
N50	279.481	tmRNA (ssrA)	1
N90	43.054	ncRNA regions	20
L50	4	oriC/oriV	1
L90	14		
GC (%)	47.82		
Coverage (10X)	99.97		
Avg. coverage depth	104

Out of 2977 CDS, 1544 (51.8%) were assigned to 23 different KEGG pathways. The most represented KOs were carbohydrate metabolism (282, 18.35%), protein families: genetic information processing (13.66%), and protein families: signalling and cellular processes (11.06%), respectively (Supplementary Table S4).

### Species Identification and Phylogenomic Analysis

Strain PRA205 was previously identified as *Lcb. casei* (formerly *Lactobacillus casei*) based on 16S rRNA gene sequencing [20]. *Lcb. casei* is closely related to other *Lacticaseibacillus* species, including *Lcb. rhamnosus*, *Lacticaseibacillus paracasei*, and the newly described species *Lcb. zeae* subsp. *zeae* [82, 83], *Lcb. huelsenbergensis* [40], *Lcb. parahuelsenbergensis*, *Lcb. styriensis*, and *Lcb. zeae* subsp. *silagei* [41]. However, 16S rRNA barcoding is insufficient to distinguish phylogenetically related species sharing more than 99.7% sequence identity.

**Fig. 2:**
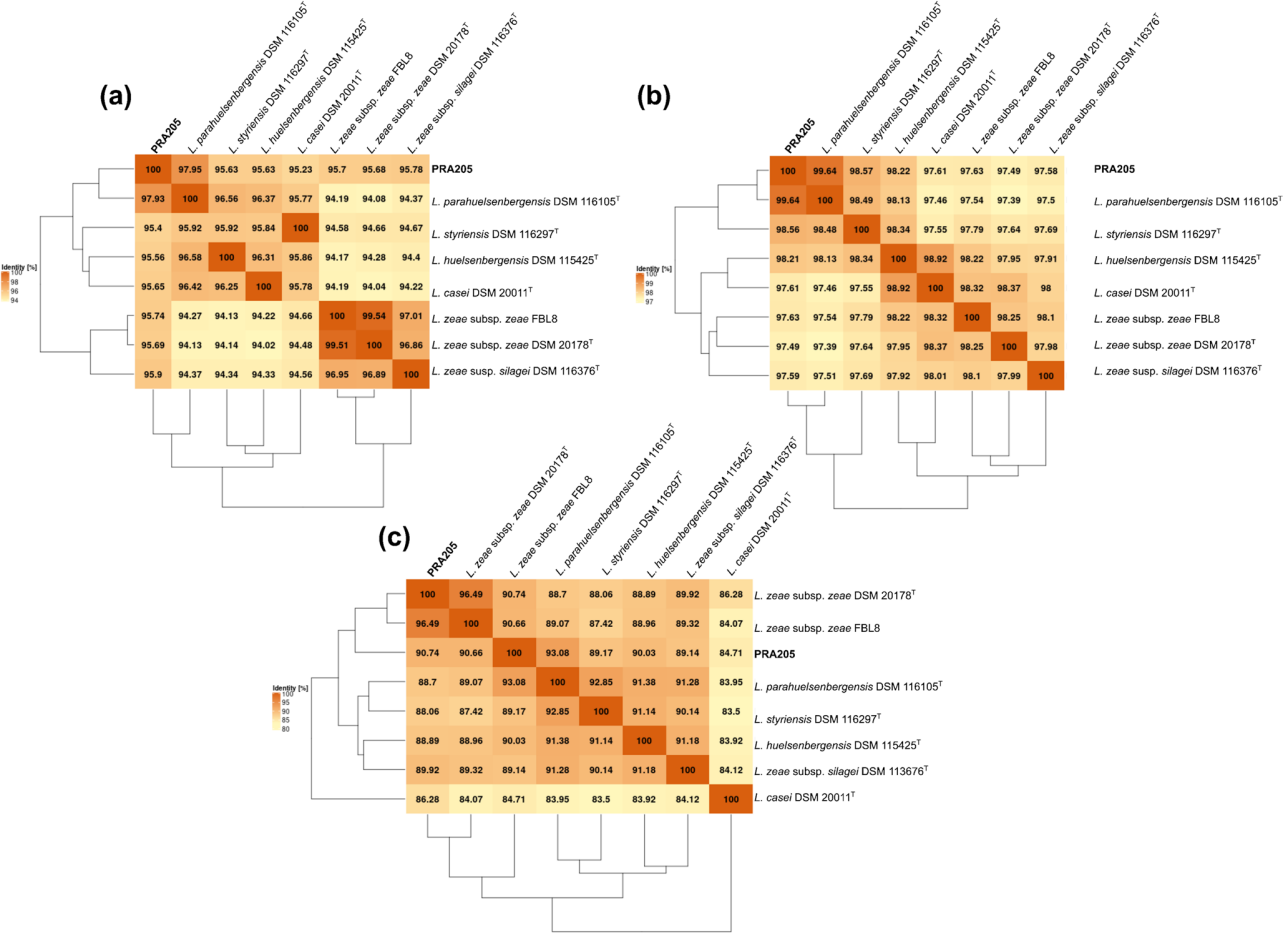
Clustering and heatmap analyses of *Lacticaseibacillus* sp. strain PRA205 and 7 close relatives based on similarity matrices of FastANI (**a**), AAI (**b**), and POCP (**c**) metrics, respectively

To clarify the classification of PRA205, we computed ANIb and ANIm values for the PRA205 genome using 11 phylogenetically related strains (Supplementary Tables S5 and S6). In two of these comparisons, ANI values exceed the 95% species threshold, making it challenging to assign PRA205 unambiguously to either *Lcb. parahuelsenbergensis* (ANIb/ANIm of 97.72%/98.07%) or *Lcb. zeae* subsp. *zeae* (ANIb/ANIm of 94.85%/95.33%). Similarly, dDDH values were 93.3% (confidence interval: 90.6–95.3%) with *Lcb. parahuelsenbergensis* DSM 116,105^T^; 82% (confidence interval: 78.2–85.1%) with *Lcb. zeae* subsp. *zeae* DSM 20,178^T^; and 69.0% (confidence interval: 65.1–72.7%) with *Lcb. casei* DSM 20,011^T^, respectively (Supplementary Table S7). Furthermore, FastANI and AAI analyses showed that strain PRA205 clustered with *Lcb. parahuelsenbergensis* DSM 116,105^T^ (Fig. 2a and b), whereas POCP metrics supported the clustering of strain PRA205 with *Lcb. zeae* subsp. *zeae* DSM 20,178^T^ (Fig. 2c). Given that species boundaries are defined by an ANI value of 95–96% and a dDDH value of 70%, we can confidently exclude *Lcb. casei* as the species designation for strain PRA205. However, the data does not allow for a conclusive assignment of PRA205 to either *Lcb. parahuelsenbergensis* or *Lcb. zeae* subsp. *zeae.*

**Fig. 3:**
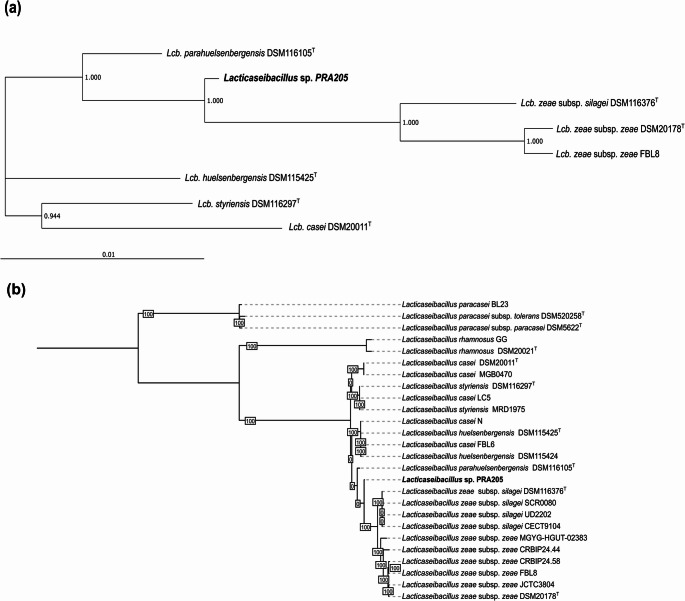
Core genome phylogenetic analyses of *Lacticaseibacillus* sp. PRA205. (**a**) Core genome phylogenetic tree depicting the relationships of strain PRA205 with 7 publicly available *Lacticaseibacillus* spp. genomes, as computed in EDGAR3.0 [54]. Reciprocal best BLAST hits against all other genomes were checked with *Lacticaseibacillus* sp. strain PRA205 as the reference genome. Alignments of each core gene set were generated with MUSCLE and concatenated [57]. A phylogenetic tree was constructed with approximately-maximum-likelihood using FastTree software (http://www.microbesonline.org/fasttree/). (**b**) Core genome phylogenetic tree depicting the relationships of strain PRA205 with 25 publicly available *Lacticaseibacillus* spp. genomes obtained with PhyloPhlAn 3 in the fast mode [58]. The final phylogenetic tree was inferred using the maximum-likelihood method with RAxML v.8.2.12 [59]. All trees were visualised using iTOL [60]

To resolve the taxonomic position of strain PRA205, two different whole-genome phylogeny construction methods were used. In the core genome-based phylogenetic analysis performed with the EDGAR3.0 pipeline, a dataset of eight *Lacticaseibacillus* genomes was used, and strain PRA205 formed a separate branch near *Lcb. zeae* subsp. *zeae* and *Lcb. zeae* subsp. *silagei*, suggesting close but distinct relatedness (Fig. 3a).

Analysis with PhyloPhlAn 3.0 included a larger dataset of 25 *Lacticaseibacillus* genomes. The resulting topology of the ML tree identified three clusters. The first cluster, referred to as *Lcb. styriensis*, consisted of *Lcb. styriensis* DSM 116297^T^, *Lcb. styriensis* MRD1975, and *Lcb. casei* strain LC5, which should be re-attributed to *Lcb. styriensis*. The second cluster, referred to as *Lcb. casei*, included the only two genomes present in GenBank (last accessed March 2025) and correctly attributed to *Lcb. casei*, namely those of *Lcb. casei* strains DSM 20011^T^ and MGB0470. The third cluster, referred to as *Lcb. huelsenbergensis*, including *Lcb. casei* strains N and FBL6, in addition to the two strains previously described as *Lcb. huelsenbergensis* such as DSM 115425^T^ and DSM 115424 [40]. Also in this case, strains FBL6 and N, previously annotated as *Lcb. casei*, could be re-attributed to the novel species *Lcb. huelsenbergensis*. According to [41], *Lcb. parahuelsenbergensis* DSM 116105^T^ branched alone. Finally, strain PRA205 was closely related but distinct from *Lcb. parahuelsenbergensis*, *Lcb. styriensis*, *Lcb. huelsenbergensis*, *Lcb. zeae*, and *Lcb. casei* (Fig. 3).

Based on these findings, strain PRA205 was strongly related to *Lcb. parahuelsenbergensis* but cannot be univocally attributed to this species.

### Genome Comparison

Comparison of PRA205 genome with those of five related species revealed that the genome size of strain PRA205 was slightly larger than that of *Lcb. casei* and *Lcb. rhamnosus* and was more comparable to those of *Lcb. zeae* subsp. *zeae* and four novel species recently isolated from silage, including *Lcb. huelsenbergensis* [40], *Lcb. parahuelsenbergensis*, *Lcb. styriensis*, and *Lcb. zeae* subsp. *silagei* [41] (Table 2). Accordingly, the CDS counts in PRA205 and in *Lcb. zeae* subsp. *zeae* DSM 20,178^T^ were higher than in other related species (Table 2).

**Table 2:** Overview of genomic features, Glycosyl transferase (GT) genes, and CRISPR-Cas systems in the genomes of strain PRA205 and its close relatives. Abbreviations: Lh, *Lcb. huelsenbergensis* DSM 115425^T^; Lph, *Lcb. parahelsenbergenesis* DSM 116105^T^; Ls, *Lcb. Sstyriensis* DSM 116297^T^; Lzs, *Lcb. zeae* subsp. *silagei* DSM 116376^T^; Lzz, *Lcb. zeae* subsp. *zeae* DSM 20178^T^; GT: Glycosyl transferase; na, not applicable

Features		PRA 205	Lzz	Ls	Lh	Lph	Lzs
Genome	Size (Mb)	3.2	2.98	2.99	2.95	2.91	2.91
	GC (%)	47.82	47.74	47.93	47.94	48.02	48.03
	CDS	2977	2961	2906	2798	2792	2777
	rRNA	6	6	15	15	15	15
	tRNA	54	53	60	59	59	59
GT	GT4	19	15	15	15	14	14
	GT2	7	7	8	7	9	7
	GT1	6	6	5	9	7	3
	GT51	3	4	2	3	3	3
	GT5	2	2	2	2	2	2
	GT28	1	0	1	1	1	1
	GT35	1	1	1	1	1	1
	GT9	1	1	0	0	1	1
	GT8	1	1	0	1	1	1
GH	GH38	-	-	+	-	-	-
*araA*		+	-	-	+	+	+
CRISPR region	N°	0	6	0	1	1	8
*cas* gene	N°	2	3	0	0	0	2
	Type	both CAS putative	CAS-TypeIC, CAS putative, CAS putative	na	na	na	CAS-TypeIC, CAS-TypeIIA_1

**Fig. 4:**
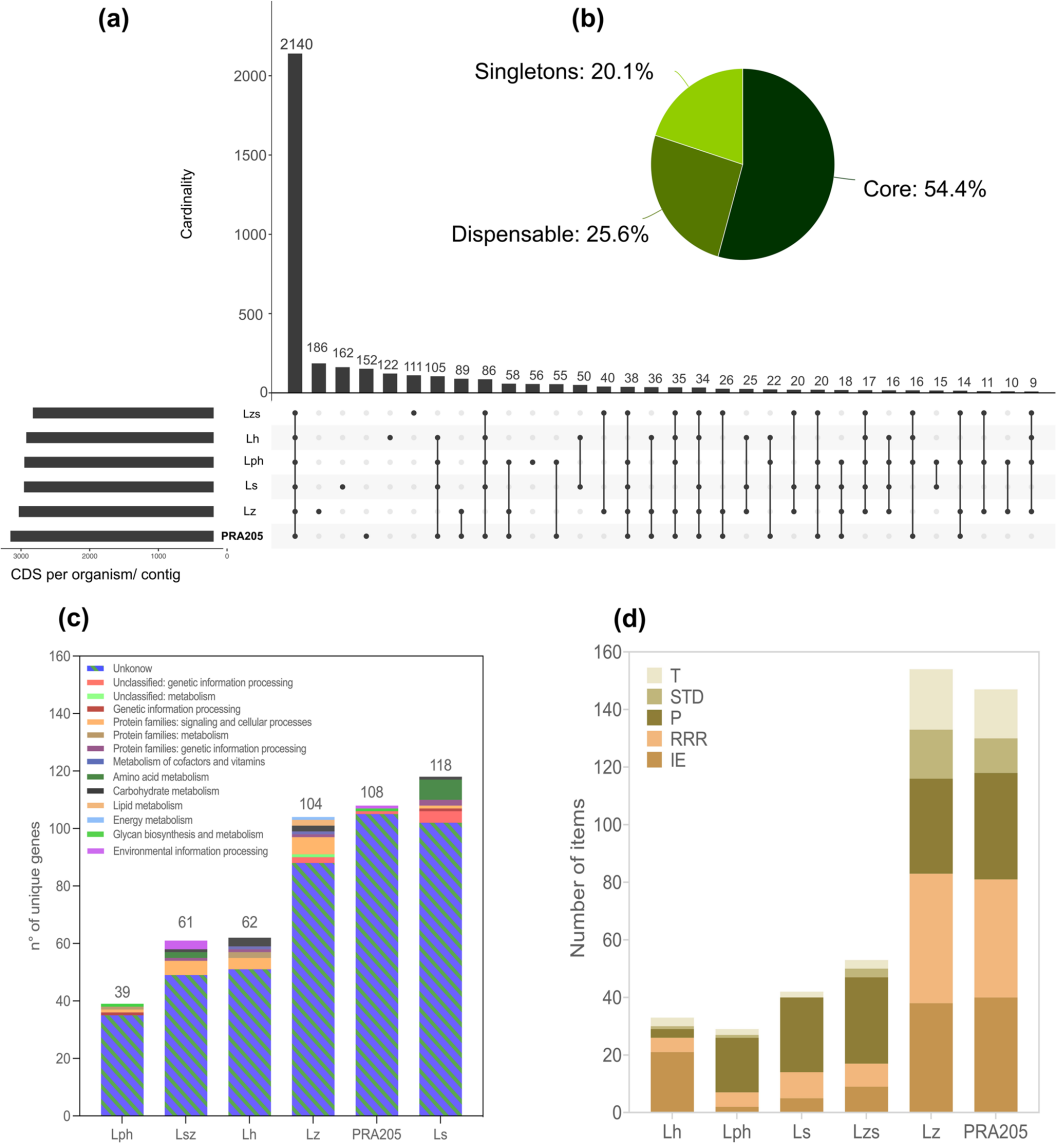
Comparative genomics analysis across 6 *Lacticaseibacillus* spp. strains, including *Lacticaseibacillus* sp. PRA205, *Lcb. zeae* subsp. *zeae* DSM 20178^T^, *Lcb. zeae* subsp. *silagei* DSM 116376^T^, *Lcb. parahuelsenbergensis* DSM 116105^T^, *Lcb. huelsenbergensis* DSM 115425^T^, and *Lcb. styriensis* DSM 116297^T^.(**a**) Upset plot showing the distribution and intersection of orthogroups across the six genomes. (**b**) Distribution of core-genome and accessory genome. (**c**) KEGG classification of unique genes. (**d**) Distribution of Genetic Mobile Elements (MGEs). Abbreviations: T, transposase; STD; stability/transfer/defence; P, phage-related genes; RRR, replication/recombination/repair; IE, integration/excision; Lh, *Lcb. huelsenbergensis*; Lph, *Lcb. parahuelsenbergensis*; Ls, *Lcb. styriensis*; Lzs, *Lcb. zeae* subsp. *silagei*; Lz, *Lcb. zeae* subsp. *zeae*

Orthology relationships among PRA205 and five related strains were assessed using the EDGAR3.0 pipeline. The pan-genome included 3930 CDS: 2136 core genes (54.4%), 789 strain-specific genes (20.1%), and 1005 dispensable genes (25.6%) (Fig. 4a). The large core genome suggests a strong conservation of essential functions across all strains despite their attribution to different species, while the high number of strain-specific genes could be a signal of niche adaptation. However, the functional roles of the strain-specific genes and their contribution to ecological niche adaptation have not yet been investigated. COG category distribution across demonstrated that most orthogroups were non-annotated, followed by orthogroups classified as carbohydrate transport and metabolism in core, dispensable, and singletons genomes (Supplementary Table S8). *Lcb. zeae* subsp. *zeae* DSM 20178^T^ had the highest number of unique genes (Fig. 4c), while PRA205 displayed 108 singletons, 105 of which are unclassified. The remaining three singletons are involved in unclassified – genetic information processing (1), environmental information processing (1), and glycan biosynthesis and metabolism (1) (Fig. 4c).

Mobile genetic elements (MGEs) contribute to genome plasticity and horizontal gene transfer (HGT) [84]. Analysis with MobileOG-db revealed that PRA205 displayed a similar MGE profile to *Lcb. zeae* subsp. *zeae* DSM 20178^T^, and a greater MGEs number than *Lcb. parahuelsenbergensis* DSM 116105^T^, *Lcb. huelsenbergensis* DSM 115425^T^, *Lcb. styriensis* DSM 116297^T^, and *Lcb. zeae* subsp. *silagei* DSM 116376^T^. Most of 147 MGE-associated regions in the PRA205 genome were involved in integration/excision, replication/recombination/repair, and phage-related genes (Fig. 4d).

### Biosafety,Prophage, and CRISPR-Cas Assessments

According to EFSA [66], the genome of the PRA205 strain was submitted to in silico biosafety assessments (Supplementary Table S9**)**. Based on AMRFinder and ResFinder analyses, strain PRA205 can be considered safe in relation to the potential dissemination of AMR genes. Neither virulence nor pathogenic genes were detected. PathogenFinder showed a probability of being a human pathogen of 0.092 (above 1), while the probiotic potential risk score (PPRS) was 2.00. These results are in accordance with the QPS status of *Lacticaseibacillus* sp. strain PRA205.

No plasmid replication initiation proteins were detected in the genome of strain PRA205 using PlasmidFinder v2.1, suggesting the absence of plasmids. Similarly, no plasmid sequences were found in the closely related species.

At least one prophage sequence was detected in each of the analysed genomes (Supplementary Table S10). All these prophages belonged to the *Siphoviridae* family [85], which is the most prevalent prophage family infecting *Lacticaseibacillus* strains [86, 87]. Specifically, strain PRA205 harboured three prophage regions, including one transposable element, whereas *Lcb. parahuelsenbergensis* DSM 116,105^T^ had only one.

Analysis of CRISPR-Cas systems showed that PRA205 genome displayed two putative *cas* clusters (13.2 kb and 3.5 kb) but no CRISPR arrays (Supplementary Tables S12 and S13). The absence of complete CRISPR-Cas systems may be due to assembly limitations from short-read Illumina sequencing, which struggles to resolve repetitive regions. Conversely, *Lcb. parahuelsenbergensis* DSM 116,105^T^ and *Lcb. huelsenbergensis* DSM 115,425^T^ had one CRISPR array each, but no *cas* genes. *Lcb. styriensis* lacked both CRISPR elements and *cas* genes. Complete CRISPR-Cas systems were found only in *Lcb. zeae* subsp. *silagei* and *Lcb. zeae* subsp. *zeae* (Table 2; Supplementary Tables S11 and S12). CRISPR-Cas systems are frequently lost or rendered inactive in bacteria undergoing extensive horizontal gene transfer (HGT) [88]. The high abundance of prophage remnants, transposases, and integrative MGEs in the PRA205 genome may therefore reflect an evolutionary trade-off between defence and genetic plasticity. Loss or inactivation of CRISPR-Cas machinery can enhance horizontal acquisition of MGEs, which, in turn, may introduce beneficial traits such as metabolic flexibility and stress tolerance. Conversely, recurrent phage infections can impose selective pressure favouring strains with compromised CRISPR-Cas systems, as active CRISPR loci may restrict beneficial phage-mediated gene flow or trigger autoimmunity through self-targeting [88]. Thus, the apparent lack of a functional CRISPR-Cas system in PRA205, coupled with its extensive MGE repertoire, suggests a genome shaped by frequent horizontal gene transfer rather than by strong antiviral defence. This interplay between MGE activity and CRISPR-Cas loss may have contributed to the adaptive evolution of PRA205 within the cheese microbiota, promoting diversification and acquisition of niche-specific traits.

### Search for Functional Traits-Associated Genes

To identify genes potentially linked to probiotic traits, we analysed the genome of strain PRA205 for biosynthetic gene clusters (BGCs), putative bacteriocins, carbohydrate-active enzymes (CAZymes), and genes involved in the acidic stress response, gastrointestinal survival, and adhesion.

antiSMASH 8.0 predicted five BGCs in PRA205 genome, namely three ribosomally encoded and post-translationally modified peptides (RiPP)-like gene clusters, one linear azol(in)e-containing peptides gene clusters, and one terpene precursor (Supplementary Table S12). The three RiPP-like clusters resembled the *Escherichia coli* BGC encoding microcin L (similarity 0.44), the *Lactobacillus gasseri* BGC encoding gassericin T/E (similarity 0.53), and the *Enterococcus faecium* BGC encoding enterocin A (0.56 similarity), respectively. Among closely related strains, *Lcb. parahuelsenbergensis* exhibited the most similar BGCs profile to PRA205 (Supplementary Table S12).

**Fig. 5:**
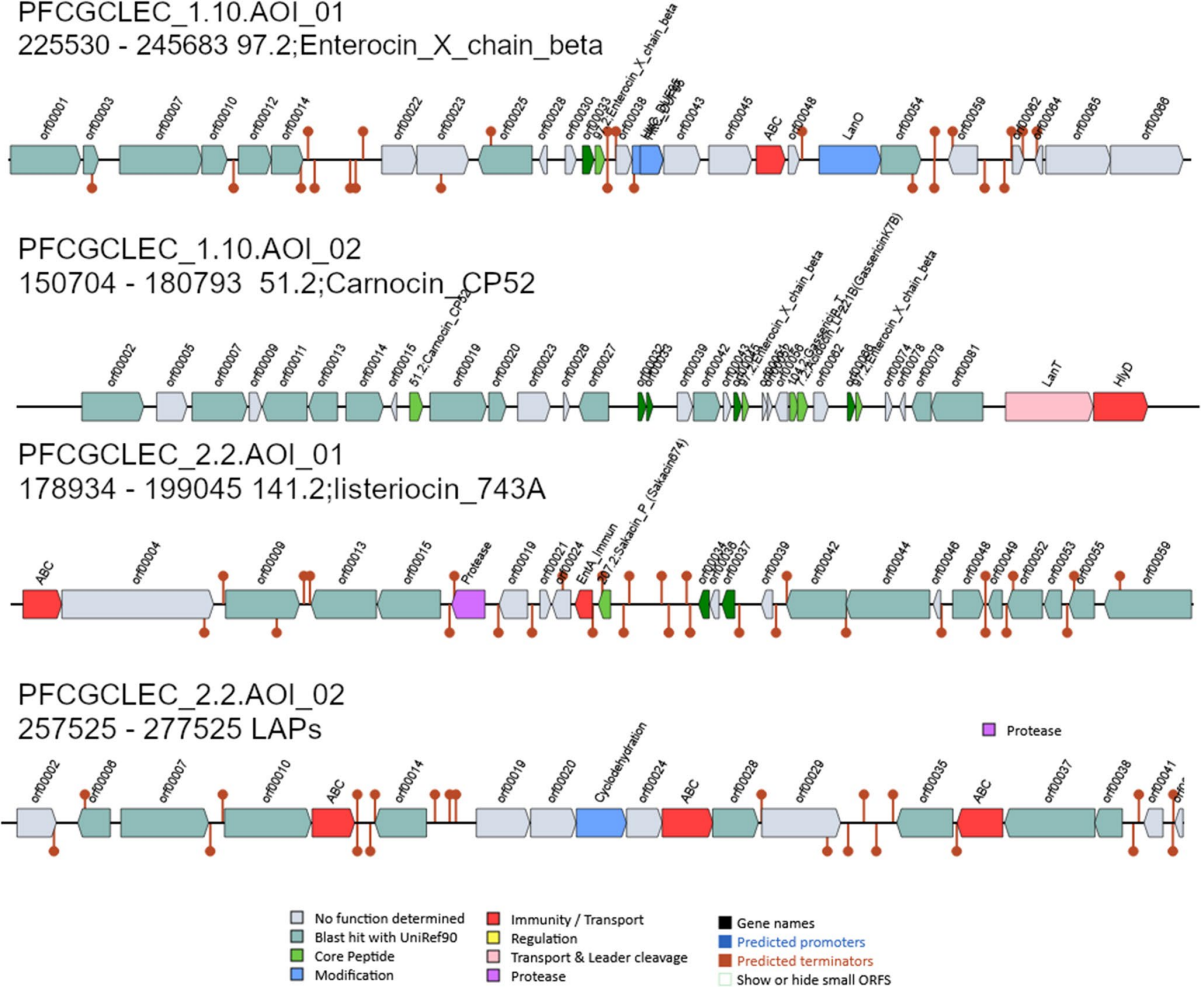
BAGEL5 graphical output for four putative bacteriocin gene clusters identified in *Lacticaseibacillus* sp. PRA205

BAGEL5 analysis identified four areas of interest (AOIs), three of which overlapped with those predicted by antiSMASH. The fourth AOI (PFCGCLEC_1.10.AOI_01) contained several genes involved in class IIc bacteriocin production (Fig. 5). In particular, the cluster included two bacteriocins: the bacteriocin at orf00033 was identified as a ComC/BlpC family leader-containing pheromone/bacteriocin (WP_138130143.1) from *Lcb. zeae*, and the adjacent downstream ORF encoded Enterocin X beta chain, identified as a bacteriocin leader domain-containing protein commonly reported in many *Lacticaseibacillus* spp. strains [89]. The presence of these putative bacteriocin gene clusters suggests that PRA205 may produce antimicrobial peptides conferring a competitive advantage within complex microbial communities, such as those found in fermented foods or the gastrointestinal tract. Such traits could enhance its ecological fitness, inhibit competing microorganisms, and support its persistence and functional contribution as a probiotic. Therefore, bacteriocin biosynthesis potential represents a valuable probiotic attribute that merits further experimental validation.

The search for CAZymes revealed that 54.40% of the carbohydrate metabolism-related genes were annotated as glycosyltransferases (GTs) and 41.33% as glycoside hydrolases (GHs) in PRA205 genome (Table 2). A similar distribution was found in *Lcb. zeae* subsp. *zeae* DSM 20178^T^ (GT: 52.11%, GH: 42.25%), *Lcb. parahuelsenbergensis* (GT: 54.17%, GH: 40.28%), and *Lcb. huelsenbergensis* (GT: 54.29%, GH: 41.43%). In contrast, *Lcb. styriensis* and *Lcb. zeae* subsp. *silagei* showed slightly lower GT levels (48.61% and 47.89%, respectively) (Table 2).

GHs play a critical role in the metabolism of complex carbohydrates, as they mainly hydrolyse glycosidic bonds and the glycosidic linkages between carbohydrates [90]. Among GHs, glycoside hydrolases of family 38 are Class II α-mannosidases involved in the hydrolysis of terminal, non-reducing α-D-mannose residues in α-D-mannosides. *Lcb. styriensis* was the only *Lacticaseibacillus* species possessing a gene encoding α-mannosidase and therefore positive for α-mannosidase activity [41]. This gene was missing in strain PRA205 (Table 2).

GTs catalyse the formation of a glycosidic bond between phospho-activated sugars and various acceptors. These enzymes are involved in stress response, biofilm formation, and the biosynthesis of exopolysaccharides (EPS), which contribute to probiotic traits such as colonisation and persistence [91]. PRA205 contained 41 GT genes from 9 families, 19 of which belonged to family 4 (Table 2). GT families 2 and 4 were most prevalent across all strains (Table 2).

Notably, the GT8 gene, proposed as a molecular marker to distinguish *Lcb. casei* from *Lcb. zeae* [92], was present in PRA205 (96.44% identity), *Lcb. zeae* subsp. *zeae*, *Lcb. zeae* subsp. *silagei*, *Lcb. parahuelsenbergensis*, and *Lcb. huelsenbergensis* but absent in *Lcb. styriensis* and *Lcb. casei* DSM 20011^T^. Analysis of pairwise sequence similarity revealed that GT8 nucleotide sequences are poorly conserved among these strains (Supplementary Table S13), suggesting that this gene may serve as a target for PRA205-specific primer design.

The *araA* gene, which encodes L-arabinose isomerase, was detected in PRA205 (Table 2). This gene is also present in *Lcb. parahuelsenbergensis*, *Lcb. zeae* subsp. *silagei*, and *Lcb. styriensis* but absent in *Lcb. zeae* subsp. *zeae* DSM 20178^T^ [40, 41]. Its presence suggests that PRA205 may metabolise arabinose, a potentially valuable probiotic trait [93].

Among the probiotic-associated traits, tolerance to acidic stress encountered during gastrointestinal transit is relevant. In our previous study, strain PRA205 was shown to withstand low pH conditions [21]. Consistent with this phenotype, PRA205 genome contained multiple genes involved in acid stress response, including those encoding the F-type proton pump (*atpA*–*atpH*), Na^+^:H^+^ antiporter (*npaA*), and ornithine decarboxylase (*odcI*) (Table 3). These systems contribute to intracellular pH homeostasis by coupling ATP hydrolysis to proton extrusion [94].

**Table 3:** Probiotic-related genes found in *Lacticaseibacillus* sp. PRA205

Phenotype	Gene ID	Name	Gene description	Position (Contig; coordinates)
Acid tolerance	PFCGCLEC_02899	*atpC*	F-type H+-transporting ATPase epsilon chain	Contig_19; 5940.6371
	PFCGCLEC_02900	*atpD*	F-type H+-transporting ATPase subunit beta	Contig_19; 6386.7852
	PFCGCLEC_02901	*atpG*	F-type H+-transporting ATPase gamma chain	Contig_19; 8028.8951
	PFCGCLEC_02902	*atpA*	F-type H+-transporting ATPase subunit alpha	Contig_19; 8963.10492
	PFCGCLEC_02903	*atpH*	F-type H+-transporting ATPase subunit delta	Contig_19; 10516.11061
	PFCGCLEC_02904	*atpF*	F-type H+-transporting ATPase subunit b	Contig_19; 11048.11536
	PFCGCLEC_02905	*atpE*	F-type H+-transporting ATPase subunit c	Contig_19; 11571.11783
	PFCGCLEC_02906	*atpB*	F-type H+-transporting ATPase subunit a	Contig_19; 11806.12516
	PFCGCLEC_02705	*napA*	Na(+)/H(+) antiporter (CPA2 family)	Contig_14; 30646.31800
	PFCGCLEC_02301	*odcI*	Inducible ornithine decarboxylase	Contig_09; 39301.41391
	PFCGCLEC_02602	*potE*	Ornithine-Putrescine antiporter	Contig_12; 23228.24583
Adhesion	PFCGCLEC_02966	*ltasS*	Lipoteichoic acid synthase	Contig_21; 12038.14134
	PFCGCLEC_01247	*tuf*	Elongation factor Tu	Contig_03; 244598.245788
	PFCGCLEC_02369	*tsf*	Elongation factor Ts	Contig_09; 106024.106905
	PFCGCLEC_00024	*fusA*	Elongation factor G	Contig_01; 28760.30862
	PFCGCLEC_02853	*dltA*	D-alanine–D-alanyl carrier protein ligase	Contig_17; 33208.34728
	PFCGCLEC_02962	*cpoA*	Alpha-galactosylglucosyldiacylglycerol synthase	Contig_21; 7736.8764
	PFCGCLEC_01140	*fbpA*	Fibronectin-binding protein	Contig_03; 126400.128100
	PFCGCLEC_00218	*-*	Hypothetical protein with MucBP domain	Contig_01; 221391.222581
	PFCGCLEC_00997	*-*	Hypothetical protein with MucBP domain	Contig_02; 377598.379850
	PFCGCLEC_02256	*-*	Hypothetical protein with MucBP domain	Contig_08; 142726.143214
	PFCGCLEC_00943	*oppA*	Oligopeptide-binding protein OppA	Contig_02; 332806.334416
	PFCGCLEC_00350	*groL*	chaperonin GroEL	Contig_01; 356658.358295

Notably, PRA205 lacks genes of the arginine deiminase (ADI) pathway as well as tyrosine-, lysine-, and histidine-decarboxylases. This indicates that, although genetically adapted to cope with acidic stress, the strain does not produce harmful biogenic amines, with the possible exception of putrescine (Table 3). The latter is generated via ornithine decarboxylation, a process generally coupled with amino acid transport by antiporter proteins [94]. This pathway contributes to cytosolic alkalinisation and proton motive force generation, which can be exploited for stress resistance and ATP production. Consistently, PRA205 harbours both the ornithine decarboxylase-encoding gene *odcI* and *potE*, which encodes a substrate/product exchanger (Table 3) [95].

Although PRA205 also tolerates bile acids [21], another stress encountered in gastrointestinal transit, its genome lacks the cholylglycine hydrolase gene (*bsh*). This finding supports the view that bile salt tolerance is a polygenic trait determined by multiple mechanisms other than *bsh* [42].

Lastly, PRA205 was shown to possess strong auto-aggregation and high cell-surface hydrophobicity, two in vitro-assessed properties that are predictive of adhesion capability in vivo [21]. This adhesive phenotype was supported by several genes implicated in binding to intestinal epithelial cells, mucin, and fibronectin, including fbpA and MucBP-domain proteins, which could mediate adhesion in vivo (Table 3) [96, 97].

### Reconstruction of the Proteolytic System

Among the functional traits of strain PRA205, the ability to release anti-hypertensive peptides from caseins has been well documented [22, 23]. To elucidate the genetic basis of this phenotype, we analysed the distribution of protease and peptidase-encoding genes in PRA205 and 12 related LAB species (Table 4).

**Table 4.**
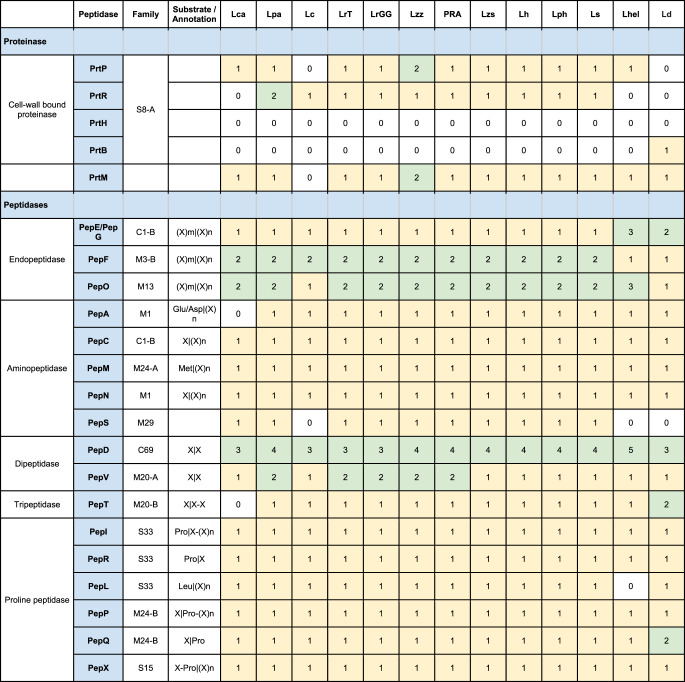
Distribution of proteinase and peptidases in the proteolytic system of *Lacticaseibacillus* species. The number of identified genes is indicated. MEROPS families are indicated. Colour shading shows the absence of a gene (white), a single gene (yellow) or multiple genes (green). The Locus_Tag codes of the genes can be found in supplementary Table S14. Abbreviations: Lca: *Lcb. casei* DSM 20011^T^; Lpa: *Lcb. paracasei* BL23; Lc: *Lcb. chiayiensis* FBL7; LrT: *Lcb. rhamnosus* DSM 20021^T^; LrGG: *Lcb. rhamnosus* GG; Lzz: *Lcb. zeae* subsp. zeae DSM 20178^T^; PRA, *Lacticaseibacillus* sp. strain PRA205; Lzs: *Lcb. zeae* subsp. *silagei* 116376 ^T^; Lh: *Lcb. huelsenbergensis* DSM 115425^T^; Lph; *Lcb. parahuelsenbergensis* DSM 116105^T^; Ls: *Lcb. styriensis* DSM 116297^T^; Lhel: *L. helveticus* DSM 20075^T^; Ld: *L. delbrueckii* subsp. *lactis* DSM 20072^T^

Consistent with previous findings in *L. helveticus* and *L. delbrueckii* subsp. *lactis* [18], protease and peptidase genes were conserved across all genomes analysed. Core peptidases—PepC, PepN, PepM, and proline-specific peptidases PepR, PepI, PepX, and PepQ—were present in single copies in all species. Gene encoding endopeptidases (PepO, PepF) and dipeptidases (PepV, PepD) were found in multiple copies in some genomes.

Strain PRA205 exhibited a complete and enriched proteolytic profile, with two copies of *PepO*, *PepF*, and *PepV* and four of *PepD* genes.A similar profile was found in *Lcb. zeae* subsp. *zeae* DSM 20,178^T^. In contrast, *Lcb. parahuelsenbergensis*, *Lcb. huelsenbergensis*, *L. styriensis*, and *Lcb. zeae* subsp. *silagei* had only one PepV-encoding gene.

Like the thermophilic species *L. helveticus*,* Lacticaseibacillus* sp. PRA205 was well-equipped to degrade proline-rich proteins like caseins as two catabolic pathways were identified in its genome: (i) PepX/PepQ, in which PepX cleaves X-Pro dipeptides, followed by PepQ-mediated hydrolysis; and (ii) PepI/PepP, where PepP releases Pro-Y-Z tripeptides, and PepI cleaves the N-terminal proline [98]. In contrast, mesophilic LAB such as *L. lactis* lack PepI and rely solely on the PepX/PepQ route [98].

In the PRA205 genome, two candidate genes encoding for an X-prolyl dipeptidyl aminopeptidase (EC 3.4.14.11) were identified: *pepX_1* (PFCGCLEC_02304; contig 9) and *pepX_2* (PFCGCLEC_02630; contig 12). Alignment of deduced amino acid sequences with the prototype *L. lactis* PepX (A0A0V8AJV2) revealed that pepX_1 contained all domains characteristic of the S15 family, namely the PepX N-terminal domain (PF09168), the catalytic S15 domain (PF02129), and the C-terminal non-catalytic domain (PF08530) (Supplementary Fig. S2). In contrast, pepX_2 lacked the N-terminal domain and resembled a CocE/Serine esterase (Q45289), indicating a misannotation. Furthermore, synteny analysis identified the *glnR* and *glnA* genes 5’-downstream of the *pepX_1* gene, consistent with the operon structure found in the close relative *Lcb. rhamnosus* [99].

These results confirm that PRA205 harbours a functional PepX-encoding gene (*pepX_1*), supporting its proteolytic activity and the functional ability to degrade caseins, releasing BPs.

### Expression Analysis of the *pepX* Gene

Peptide-rich media downregulate proteolytic genes in several LAB species, mediated mainly by the CodY regulator, which responds to intracellular branched-chain amino acids levels by binding conserved motifs in promoters of nitrogen metabolism genes (e.g., *prt*,* pepN*,* pepC*,* opp-pepO1*) [25, 100, 101]. In *L. lactis*, CodY represses *pepX* in the presence of peptides, while in *L. delbrueckii* subsp. *bulgaricus*, its regulation is completely CodY-independent [102, 103].

**Fig. 6:**
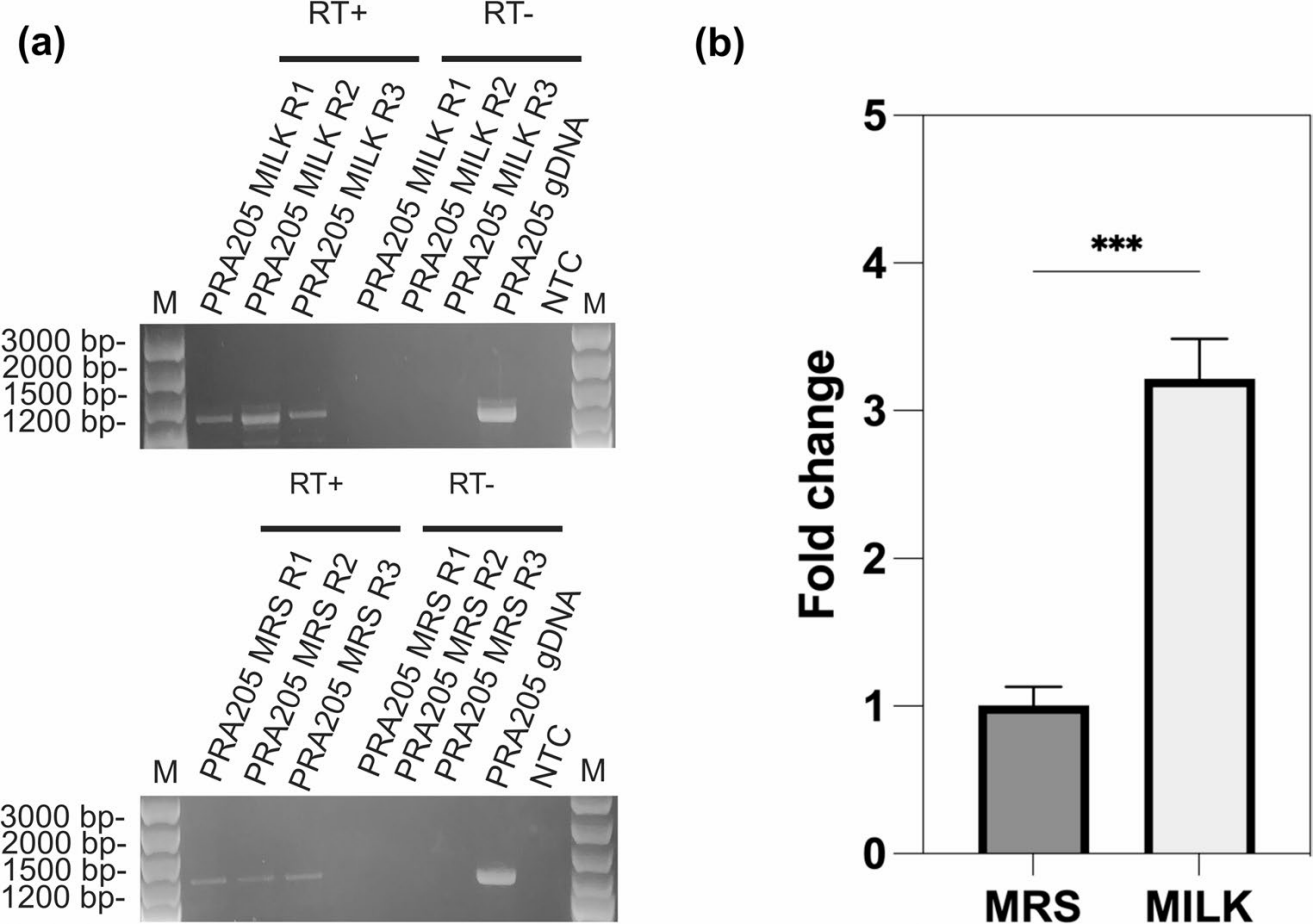
Gene expression analysis of *pepX_1* gene in *Lacticaseibacillus* sp. PRA205. (**A**) RT-PCR analysis of *pepX_1* expression in PRA205 cells grown on milk and MRS medium. The figure shows amplified cDNA products from three biological replicates; gDNA amplification was used as a positive PCR control. RT + and RT- indicate reactions with and without reverse transcription, respectively. Abbreviations: M, DNA ladder used as molecular size marker; NTC, no template control. (**B**) *pepx_1* expression level assessed by RT-qPCR assay in PRA205 grown on milk and MRS medium. Data represent the mean ± standard deviation of at least three biological replicates. Statistical differences were evaluated using Student’s t-test (*p* < 0.05); *** indicates *p* < 0.001

In strain PRA205, *prt* was completely silenced in MRS medium but expressed in a milk-like medium [25]. To investigate *pepX* gene expression, both RT-PCR and RT-qPCR were performed under control (MRS medium) and milk conditions. RT-PCR showed *pepX* transcription in both conditions, with stronger signals observed in milk (Fig. 6a). Consistently, RT-qPCR confirmed that *pepX_1* was constitutively expressed in MRS medium but significantly upregulated in milk, indicating induction under amino acid-limited conditions (Fig. 6b).

Taken together, these results suggest that, unlike *prt*, *pepX* in PRA205 is not fully repressed in peptide-rich environments, pointing to a regulatory mechanism that is at least partially independent from CodY regulator.

### PepX Partial Purification

To link genomic data to enzymatic function, the prolyl-dipeptidyl aminopeptidase activity in *Lacticaseibacillus* sp. PRA205 was assessed on cytoplasmic extract obtained from cells grown on milk by using the specific substrate Gly-Pro-pNA. The specific prolyl-dipeptidyl aminopeptidase activity was found to be 2.78 ± 0.3 U/mg of proteins. Considerable variation has been found for the prolyl-dipeptidyl aminopeptidase activity of *Lacticaseibacillus* spp. strains. Depending on the strain, prolyl-dipeptidyl aminopeptidase activity ranged from 2.4 to 50 U/mg of protein [81, 103].

Purification of PepX from the cytoplasmic extract of *Lacticaseibacillus* sp. PRA205 was carried out as reported in [103]. Gel filtration chromatography (Supplementary Fig. S3) revealed that PepX from strain PRA205 exists predominantly as a monomer, with an estimated molecular mass of approximately 80 kDa. This observation is consistent with previous reports showing that PepX occurs in a monomeric, catalytically active form (70–90 kDa) in several strains of *L. helveticus* and *L. delbrueckii*, as well as in *L. lactis* subsp. *cremoris* and *Lcb. casei* [15, 27, 103, 104]. In contrast, PepX had been purified as a homodimer of about 170–200 kDa in *L. acidophilus*, *L. delbrueckii*, *L. curvatus*, *L. helveticus*, and *L. lactis* subsp. *lactis* [15, 105, 106]. A summary of the purification details for PepX from *Lacticaseibacillus* sp. PRA205 is provided in Supplementary Table S15.

### PepX Characterisation

To define the biochemical characteristics of the purified enzyme, the effects of pH, temperature, and inhibitors were assessed using the specific substrate Gly-Pro-*p*NA.

**Fig. 7:**
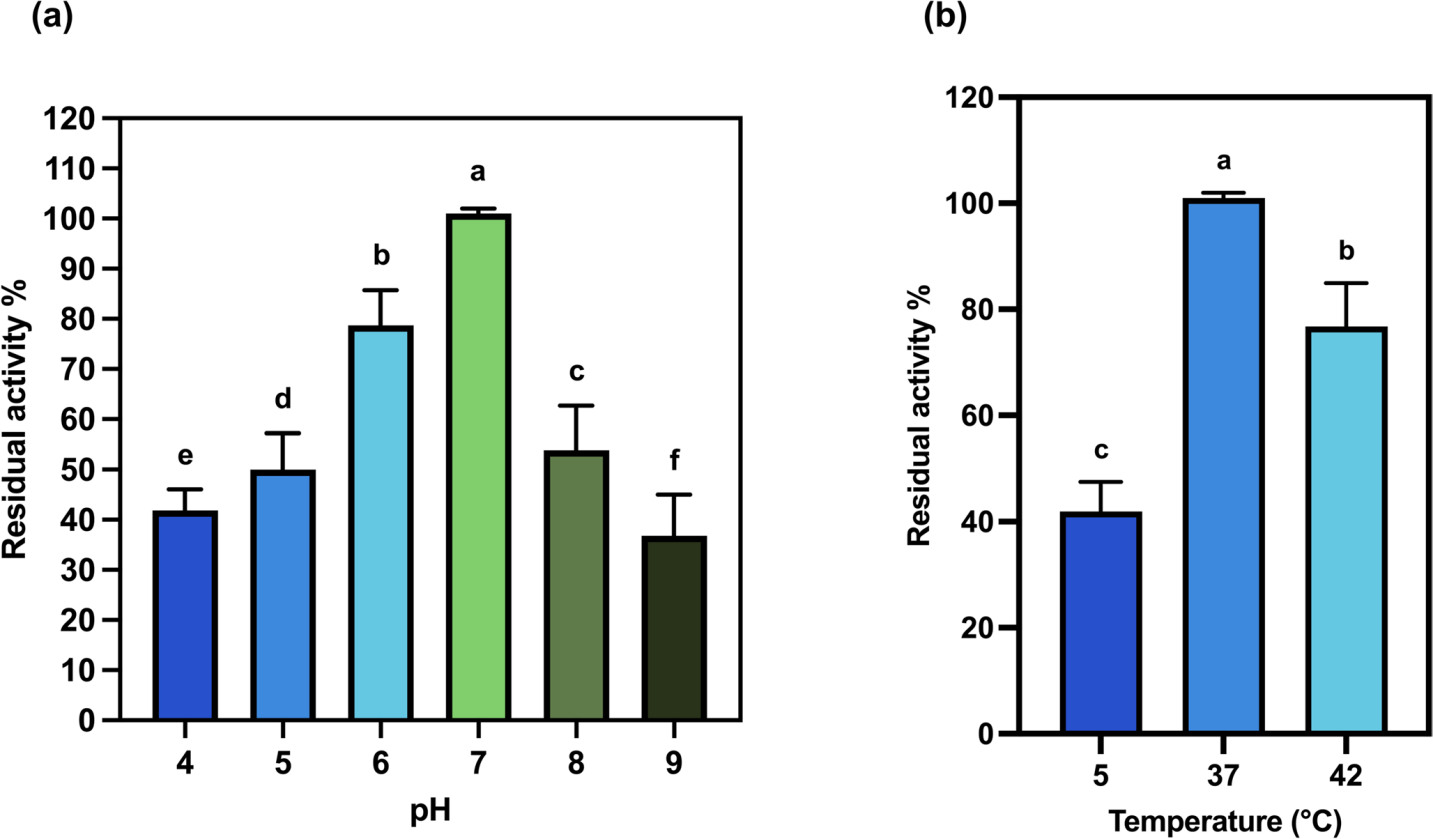
Effect of different pH values (**a**) and temperature (**b**) on PepX activity. PepX activity (expressed as a percentage) was quantified using the substrate Gly-Pro-pNA. Residual activity values were calculated based on 100% activity obtained at pH 7 and 37 °C. Different letters mean that the values are significantly different (*p* < 0.05)

The enzyme showed high activity over a wide pH range, from 6.0 to 8.0, with optimum activity at pH 7 (Fig. 7a). Values of pH optima for PepX between 6.0 and 8.0 have already been published [103–105]. A significant reduction of about 64% of PepX activity was observed at a pH value of 9. Interestingly, at pH 4.5, typical of fermented dairy products, the enzyme retained about 40% of its original activity.

The temperature dependence of *Lacticaseibacillus* sp. PRA205 PepX activity was assessed at 5 and 42 °C, which are the typical temperatures of yoghurt fermentation and fermented dairy food storage, respectively (Fig. 7b). Data showed that, at the typical yoghurt fermentation temperature of 42 °C, PepX retained approximately 77% of its activity compared with the temperature of 37 °C. Similarly, the residual activity at 5 °C was 47%. These data implied that PepX remains active during yoghurt production and at the low temperatures during cold storage.

**Fig. 8:**
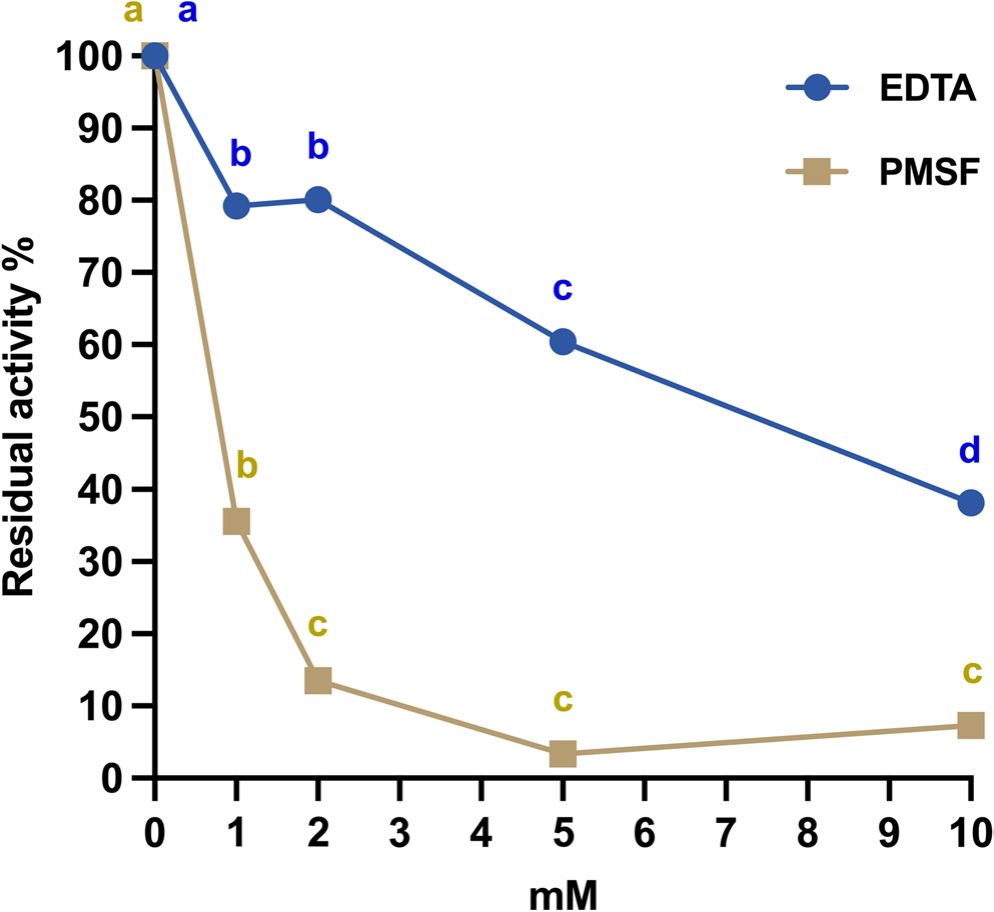
Effect of PMSF and EDTA on PepX activity. PepX activity (expressed as a percentage) was quantified using the substrate Gly-Pro-*p*NA. Residual activity (expressed in % and calculated with respect to the control reaction without inhibitors) was determined at increasing concentrations (mmol/L) of the inhibitors EDTA and PMSF. Different letters indicate significantly different values (*p* < 0.05)

The assays carried out with the partially purified enzyme in the presence of PMSF and EDTA confirmed the serine peptidase nature of the enzyme (Fig. 8). PMSF decreased the enzymatic activity by more than 40% at a concentration of 1 mmol/L. In contrast, the enzymatic activity was completely abolished at PMSF concentrations of 5 and 10 mmol/L. EDTA at 1 mmol/L had no effect on enzymatic activity, whereas a residual activity of 40% was observed at 10 mmol/L, suggesting the requirement for metals for PepX catalytic activity.

### Degradation of Bioactive Peptides by Partially Purified PepX

Considering the cleavage specificity of PepX and the presence of proline residues in many dairy-derived BPs, the ability of PepX to hydrolyse selected BPs was evaluated. Selected BPs were well-known effective inhibitors of the enzymes ACE and di-peptidyl-peptidase-IV (DPP-IV). The lactotripeptides VPP and IPP (derived from the hydrolysis of β-casein) are potent ACE inhibitors and have been effective in vivo in reducing blood pressure in both rats and humans [8]. These two peptides have been identified in several fermented dairy products such as yoghurt and cheeses [8]. The other peptides, such as LPPT, APFPE, PPF, and IPPL, have been identified as anti-diabetic peptides that are potent DPP-IV inhibitors [81].

**Fig. 9:**
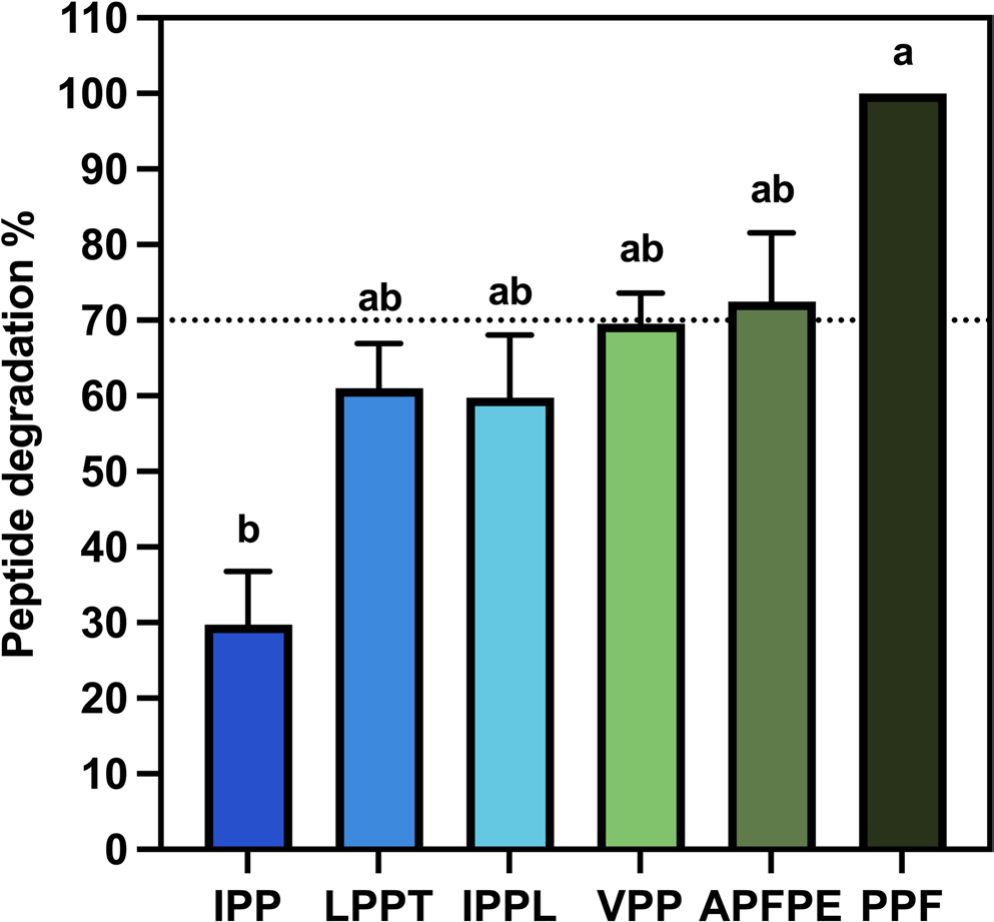
Degradation of the bioactive peptides IPP, VPP, LPPT, APFPE, PPF, and IPPL by PepX. Data are expressed as residual percentage of the peptide calculated by comparing the area under the peak (AUP) obtained from peptides incubated for 24 h with PepX and peptides incubated in the same condition but without PepX (control reaction). AUP was calculated by integrating the area under the peak, measured from the extracted ion chromatograms (EIC) obtained for each peptide (tolerance ± 5 ppm). Different letters indicate significantly different values (*p* < 0.05)

As reported in Fig. 9, all the tested peptides were cleaved by partially purified PepX, although with different extents. The highest degradation rate was observed for PPF, VPP and APFPE, whereas the lowest one was for IPP.

## Conclusions

In this study, the genetic basis of the proteolytic phenotype and high BPs-releasing activity of strain PRA205 was elucidated. Phylogenomic analyses indicated that strain PRA205 is closely related yet distinct from *Lcb. parahuelsenbergenis*, supporting its classification as a new species. Owing to the limited availability of isolates and biochemical data, its species designation remains unresolved.

The probiogenomics approach demonstrated that PRA205 carries a complete proteolytic system, encompassing both the PepX/PepQ and PepI/PepP pathways, which collectively enable efficient dual-casein hydrolysis and the consequent release of BPs. The genome also contains determinants associated with safety and probiotic potential, including bacteriocin production, acid stress tolerance, and adhesion. Functional validation of these probiotic features requires targeted in vitro and in vivo assays.

Gene expression analysis revealed a partial casitone-independent regulation of *pepX_1* gene, likely contributing to the pronounced proteolytic aptitude of PRA205. The corresponding PepX enzyme was characterised as a monomeric serine-protease of approximately 80 kDa, exhibiting activity at low temperatures and low pH conditions. PepX exhibited a dual role both in the formation and degradation of BPs during milk fermentation and dairy product storage. Overall, these findings demonstrated the potential of PRA205 as a functional dairy culture capable of modulating peptide profiles and enabling controlled BP release in fermented dairy matrices.

## Supplementary Information

Below is the link to the electronic supplementary material.

Supplementary Material 1 (DOCX. 1.05 MB)

Supplementary Material 2 (XLSX. 125 KB)

## Data Availability

This Whole Genome Shotgun project has been deposited at DDBJ/ENA/GenBank under the accession JBQQCW000000000 (BioProject: PRJNA1308501; Biosample: SAMN50695984).
