# Supplementary material for: Genomic and Proteolytic Profiling of Lacticaseibacillus sp. PRA205: Insights into PepX-Mediated Bioactive Peptide Metabolism
Source: Probiotics Antimicrob Proteins. 2025 Dec 23;18(4):6242–63. doi: 10.1007/s12602-025-10879-7 (PMC13342036; doi:10.1007/s12602-025-10879-7)
Supplement: Supplementary file 1 — Supplementary Material 1 (DOCX. 1.05 MB) [file 12602_2025_10879_MOESM1_ESM.docx]

**Genomic and Proteolytic Profiling of *Lacticaseibacillus* sp. PRA205: Insights into PepX-Mediated Bioactive Peptide Metabolism**

Marianna Cristofolini^1^, Alice Cattivelli^2^, Alessandra Barbieri^1^, Giulia Zaccarini^1^, Loris Bertoldi^3^, Lisa Solieri^1*^, Davide Tagliazucchi^2^

^1^ Lactic acid bacteria and Yeast Biotechnology Lab (LYB), Department of Life Science, University of Modena and Reggio Emilia, 42122 Reggio Emilia, Italy

^2^ Nutritional Biochemistry Lab, Department of Life Science, University of Modena and Reggio Emilia, 42122 Reggio Emilia, Italy

^3^ BMR Genomics Srl, 35131, Padua, Italy

***Corresponding author:** Lisa Solieri, Department of Life Science, University of Modena and Reggio Emilia, Via Amendola 2, Besta Building, 42122 Reggio Emilia, Italy. Email: [lisa.solieri@unimore.it](mailto:lisa.solieri@unimore.it); Phone: +39 0522 522026.

**Supplementary Information**

## 1. Materials and Methods

### 1.2 RNA extraction and Reverse Transcription PCR (RT-PCR) and qPCR (RT-qPCR) analyses

RNA was extracted from approximately 2 x 10^8^ cells using the Zymo Direct-zol RNA MiniPrep kit (Cat. No. R2071, Zymo Research, Irvine, CA, USA) with a few modifications to the manufacturer’s instructions. Briefly, after adding up to 700 µL of the Tri reagent, the mechanical lysis of cells was achieved using a Vortex Genie 2 instrument (Mo Bio Laboratories Carlsbad, CA, USA) by performing two rounds of 20 min at the highest speed alternating with 3 min on ice. The quantity of total RNA was measured spectrophotometrically using a Nanodrop Nd 1000 system (Nanodrop Technologies, Wilmington, DE, USA), and only samples with A260/280 absorbance ratio between 1.8 and 2.2 were considered for further analyses. The integrity of the total RNA was evaluated by denaturing gel electrophoresis on a 0.9% (w/v) agarose gel with formaldehyde (10 mL of 10× 3-morpholinepropane sulfonic acid [MOPS] running buffer) and 18 mL of 37% formaldehyde (12 mol/L) in pH 7.0 1× MOPS running buffer (0.4 mol/L MOPS, 1 mol/L sodium acetate, and 0.01 mol/L EDTA) after the RNA treatment at 65 °C for 10 min. To remove any contamination of gDNA, 1 µg of the RNA sample was treated with dsDNase (Cat. No. EN0771, Thermo Fisher Scientific) (final volume 40 µL) and, thereafter, RNA was reverse transcribed to cDNA at 42°C for 60 min with random hexamers (Cat. No. SO142; Thermo Fisher Scientific) and oligo (dT)18 primers (Cat. No. SO131; Thermo Fisher Scientific) using the RevertAid RT Kit (Cat. No. EP0441; Thermo Fisher Scientific) according to the manufacturer’s instructions.

The end-point RT-PCR amplification of the *pepX* gene was carried out with a Dream Taq DNA polymerase (Cat. No. EP0712; Thermo Fisher Scientific). RT-PCR of the 16S rRNA gene was used as a positive control and carried out as previously reported [1]. All RT-qPCR reactions were done in a 96-well plate using the PowerUp SYBR Green Master Mix (Cat. No. A25742; Thermo Fisher Scientific) on a QuantStudio 3 real-time PCR system (Thermo Fisher Scientific, Waltham, MA, USA). 16S rRNA was selected as a housekeeping gene. Each reaction was prepared in a 20 μL mixture containing 10 μL of the Power SYBR Green master mix, 0.3 µM of each primer, and 5 μL of properly diluted cDNA (5 µg/μL). The thermal conditions were as follows: 50°C for 2 min, 95 °C for 2 min, 40 cycles at 95 °C for 15 s, and then at 60 °C for 1 min with fluorescence measurement, and the melt curve stage including 95°C for 15 s, 60 °C for 1 min, and increasing the temperature step to 95 °C at a rate of 0.15°C/s.

### 1.2 PepX partial purification

For the partial PepX purification, a column for molecular exclusion chromatography packed with Sephadex G-100 resin, with a fractionation range between 5 and 150 kDa, was prepared and then conditioned with a Tris-HCl 50 mM buffer at pH 7.5 containing NaCl 0.1 M. The column was previously calibrated by eluting 4 standard components of known molecular weight (bromophenol blue, ferritin, bovine serum albumin and chymotrypsin). The cytoplasmic extract was first concentrated about 4 times by ultrafiltration with 30 kDa filters, centrifuging at 8360 rpm for 10 min at 4 °C to obtain an optimum separation into small volumes. Elution was carried out under isocratic conditions using the same conditioning buffer, namely Tris-HCl 50 mM at pH 7,5 containing NaCl 0.1 M. All eluted fractions were subjected to spectrophotometric reading at 280 nm and 410 nm for the presence of proteins and specific reaction catalyzed by PepX using Gly-Pro-*p*NA as substrate. Subsequently, the fractions positive for enzyme activity were collected, joined and subjected to an ultrafiltration process as described above, to concentrate the sample and remove the sodium chloride contained in the elution buffer which would interfere with the subsequent analysis.

## 2. Supplementary Figures

**Supplementary Fig. S1**. Functional distribution of core, dispensable, and singleton genes in COG categories. The dataset included *Lacticaseibacillus* sp. strain PRA205, *Lcb. zeae* subsp. *zeae* DSM 20178^T^, *Lcb. zeae* subp. *silagei* DSM 116376T, *Lcb. parahuelsenbergensis* DSM 116105^T^, *Lcb. huelsenbergensis* DSM 115425^T^, and *Lcb. styriensis* DSM 116297^T^.


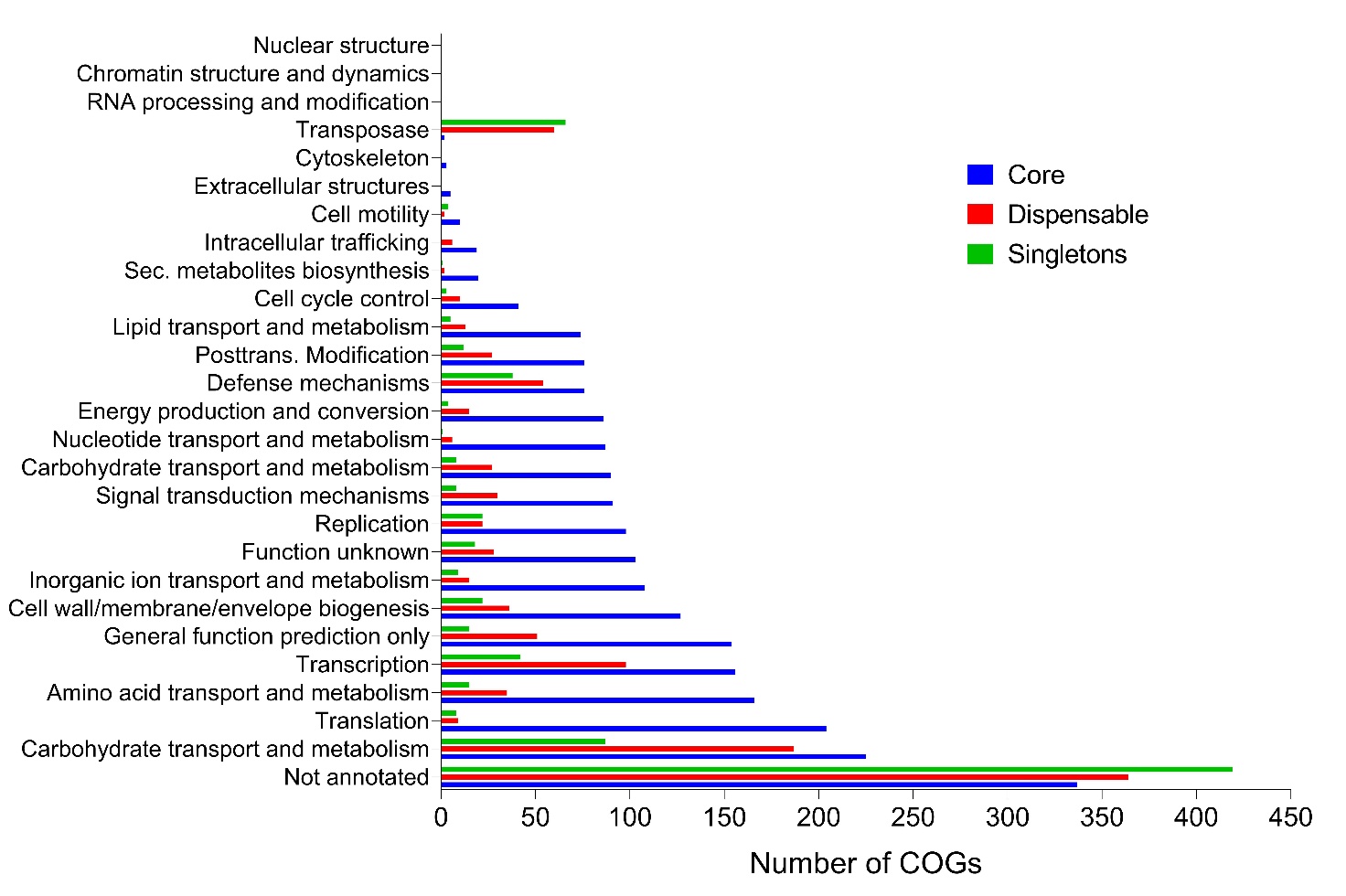


**Supplementary Fig. S2**. Comparison of PepX_1 and PePX_2 proteins. (a) Protein alignment of PepX_1 and Pep_X2 was performed with Muscle [1] and visualized with Jalview [2]. (b) Domain structures of the PepX candidate proteins. The codes denote the PFAM (PF) domain identifiers.


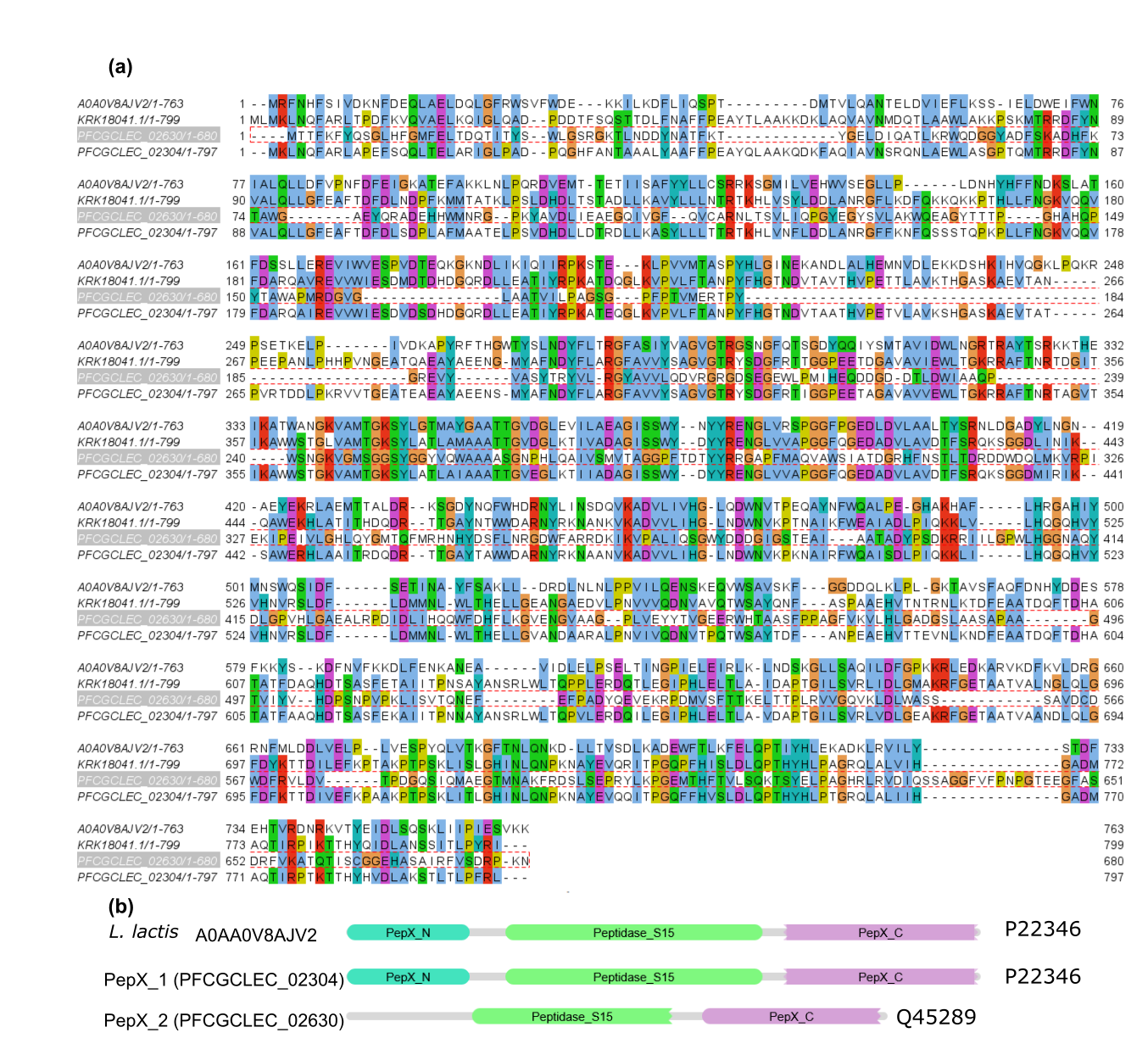


**Supplementary Fig. S3**. Partial purification of PepX from the cytoplasmic extract of PRA205 by size-exclusion chromatography (Sephadex G-100). Protein content was monitored by determining the absorbance values at 280 nm, whereas PepX activity was calculated by using Gly-Pro-*p*NA as substrate and measuring the absorbance at 410 nm.


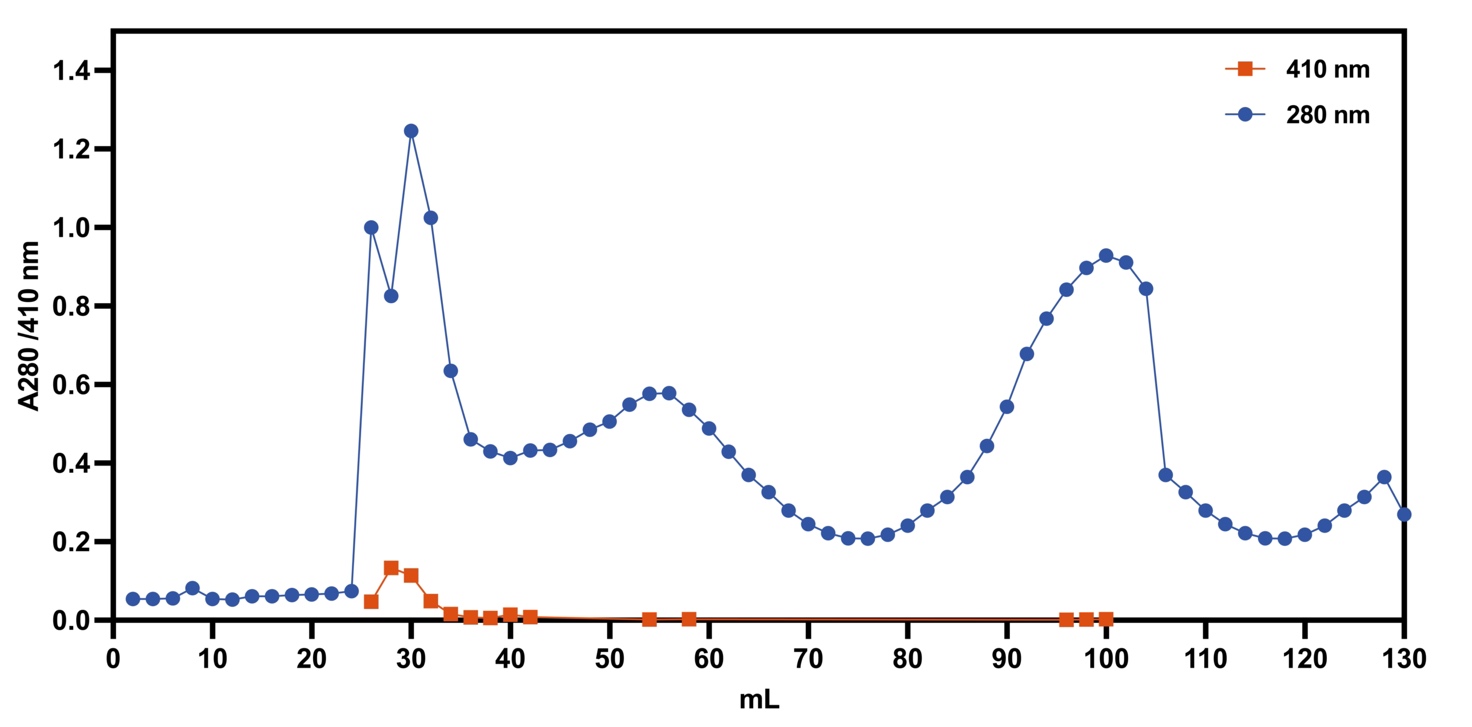


## 3. References

1. Waterhouse AM, Procter JB, Martin DMA, Clamp M, Barton GJ (2009) Jalview Version 2—a multiple sequence alignment editor and analysis workbench. Bioinformatics 25:1189–1191
2. Edgar RC (2004) MUSCLE: multiple sequence alignment with high accuracy and high throughput. Nucleic Acids Res 32:1792–1797. https://doi.org/10.1093/nar/gkh340
